# Detecting genetic heterogeneities in response to trauma: The case of 9/11

**DOI:** 10.1016/j.ssmmh.2021.100044

**Published:** 2021-12-08

**Authors:** Shiro Furuya, Jason M. Fletcher, Zijie Zhao, Zhongxuan Sun, Qiongshi Lu

**Affiliations:** a University of Wisconsin-Madison, Department of Sociology, USA; b University of Wisconsin-Madison, La Follette School of Public Affairs, USA; c University of Wisconsin-Madison, Department of Biostatistics and Medical Informatics, USA

**Keywords:** Diathesis stress model, Genetic screening, United States, Young adult, Variance polygenic score, Regression discontinuity design

## Abstract

The current study evaluates genetic heterogeneities in response to trauma among U.S. young adults. Using Add Health Wave III, which coincidently overlapped with the September 11 attacks, and a depression mean and variance polygenic scores, we investigate how the polygenic scores moderate the causal effect of 9/11 on mental health. Our results show the presence of genetic heterogeneity, where those with high genetic plasticity experience an increase in depressive symptoms following trauma while those with low genetic plasticity do not. While the documented differences in reactions to trauma are important, we also note our ability to predict responses based only on genetic measures are too imprecise to identify susceptible clinical patients. We, therefore, contend that the expected benefits from genetic screening to identify susceptible individuals after trauma are limited. Our results provide novel evidence of a specific source of an additional heterogeneity contributing to the inequality of health following trauma.

## 1. Introduction

Abundant empirical evidence links mental health and the exposure to trauma, such as experiences of domestic violence (Fergusson et al., 2005), divorce (Kim, 2011; Simon, 2002; Strohschein, 2005), racial discrimination (Burt et al., 2012; Monk, 2015), and incarceration (Sugie & Turney, 2017). Some individuals, but not all, develop depressive symptoms after trauma (Bonanno, 2004; Bonanno et al., 2011). Such heterogeneity in response to trauma has been documented in many social, environmental, and demographic factors, and scholars, especially psychologists, have examined different predictors of the consequence after trauma (Bonanno et al., 2011).

Yet, the consequences of trauma may also be predicted by genetic factors. With the rise and availability of genetic data, the hypothesized associations between genotype and depressive symptoms have begun to be examined. Levine et al. (2014) found that a polygenic measure of depressive symptoms, an indicator of cumulative impact of genotype, was significantly associated with depressive symptoms, whereas individual genetic variants (i.e., single nucleotide polymorphisms, SNPs) have been difficult to uncover. This finding is a breakthrough in the discussion of the association between genotype and depressive symptoms because it provides empirical evidence for polygenicity of depressive symptoms, rather than the presence of a single-determinant gene of depressive symptoms. Along with polygenicity of depressive symptoms, polygenicity of posttraumatic stress disorder after trauma is also documented well (Duncan et al., 2018; Gelernter et al., 2019; Nievergelt et al., 2019). Overall, research findings examining the association between genotype and depressive symptoms and mental health raise the possibility of genetic heterogeneities in response to trauma, which posits that the level of mental health responses toward trauma may also be predicted by genotypes.

Despite the presence of empirical evidence for genetic heterogeneities in response to trauma, existing research is limited in several important ways. First, most research examining genetic heterogeneities in response to trauma has been unable to capture the *immediate* response to trauma. Given that most individuals do not develop depressive symptoms at all or eventually recover to the normal level after trauma (Bonanno, 2004; Bonanno et al., 2011), the failure to measure depressive symptoms shortly after a traumatic event may lead researchers to “miss” these heterogeneous effects. This is an important limitation in light of empirical evidence for the association between short-term increase of depressive symptom and long-term poor health outcomes (Domingue et al., 2020). Second, few studies examining gene-environmental interaction utilize (quasi-)randomized environmental settings. This is a critical limitation given the likelihood of gene-environment correlation—that some genotypes place individuals at higher risk of exposure to trauma. Third, prior research has not incorporated the recent development of genetics measuring plasticity/sensitivity to environments. This limitation is critical because conventional genetic markers target the prediction of the level of an outcome but not the sensitivity to an environmental shock. This has the implication that the standard use of (mean-based) polygenic scores rather than sensitivity (variance-based) polygenic scores may lead to false negative findings of gene-environment interaction. Finally, little attention has been paid to genetic heterogeneities in response to trauma among young adults.

Evaluating genetic heterogeneities in causal effects of trauma on mental health is an important task for social scientists given an increasing interest in genetic screening to maximize the benefits of policy interventions. However, genetic screening may be premature if there are not large and predictable differences in trauma response based on genotype. A goal in this paper is to provide empirical evidence for genetic heterogeneities in response to trauma using an established quasi-experimental design.

### 1.1. Variations in response to trauma

**Fig. 1.**
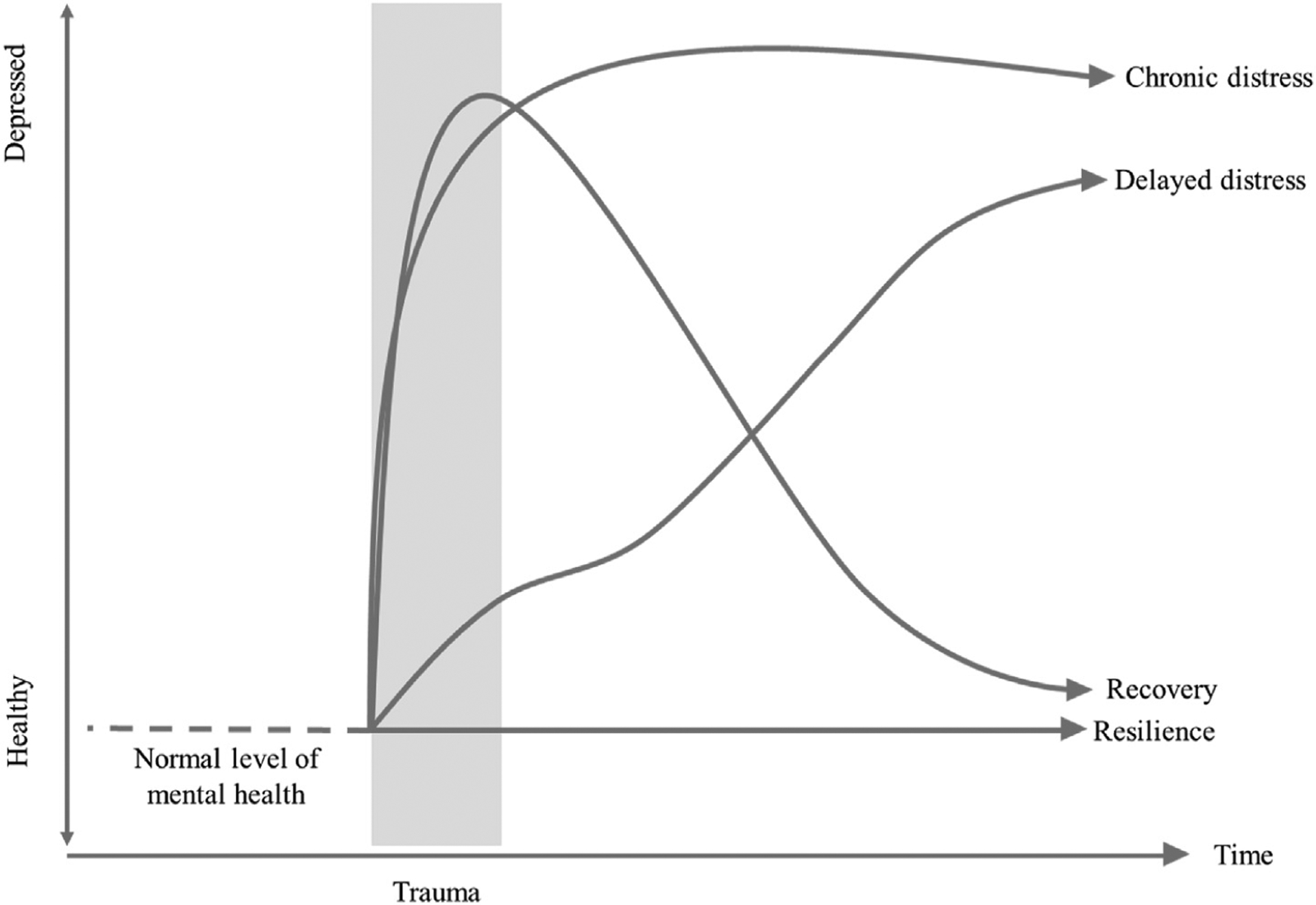
Four prototypes of the trajectory of response to trauma. Authors created the figure based on Galatzer-Levy et al. (2018)

A standard theoretical framework from psychology suggests four different trajectories of impairment after trauma —resilience, recovery, delayed distress, and chronic distress (Bonanno et al., 2011). As Fig. 1 shows, resilience is characterized by no impairment and shows a stable trajectory at the normal level. It is important to note that, in some literature, resilience is defined as not only the absence of impairment but also the presence of positive developmental outcomes following a stressful life event (Masten, 2001); however, resilience in our definition only reflects the trajectory of impairment after trauma. By contrast, recovery shows elevated depressiveness after trauma and a gradual decline to the normal level. Unlike resilience and recovery, the delayed and chronic distress trajectories have elevated depressive symptoms. In the delayed distress model, depressive symptoms moderately elevate after trauma and gradually increase over time. Chronic distress shows a sharp increase of depressive symptoms after trauma that remains constant over time.

Differences in response to trauma reflect many factors. Bonanno and colleagues (Bonanno et al., 2011) proposed personality, demographic characteristics, proximity to an exposure (i.e., trauma per se or secondary exposures), social and economic resources, past and current stress, a priori worldviews, and emotions as predictors of the response to trauma. Although results are mixed in some domains, empirical evidence has shown that a large set of characteristics predicts the response to trauma, such as male gender (Bonanno, Galea, Bucciarelli, & Vlahov, 2007), greater education (Bonanno et al., 2007), and more economic resources (Brewin et al., 2000; Norris et al., 2002).

In addition to the above-listed predictors, genotype could also be a predictor of differences in response to trauma. One theoretical framework, the diathesis-stress model, proposes that individuals carrying a higher genetic risk to develop mental problems are more likely to suffer from psychological disturbance when exposed to trauma, whereas others carrying a lower genetic risk are less likely to be affected when exposed to the same trauma (Monroe & Simons, 1991). Domingue et al. (2017) exemplified this pattern by using the (mean) depression polygenic score, which is constructed from the estimated effects of multiple genetic variants to predict traits for an individual (Young et al., 2019). Domingue and colleagues (2017) showed that older U.S. residents carrying a higher depression polygenic score have more severe depressive symptoms after experiencing a spousal death compared to those carrying a lower depression polygenic score.

While the conventional polygenic score (we label as “mean polygenic score”) targets predicting levels of an outcome, a recent methodological advancement allows us to capture sensitivities of an outcome towards environmental exposures (Johnson et al., 2021; Miao et al., 2021), a “variance polygenic score.” This methodological advancement is particularly important, as shown by prior studies that found genetic heterogeneities in effects of unemployment on health and health behaviors (Schmitz et al., 2021) and effects of education policy reform on educational attainment and health (Johnson et al., 2021) with variance polygenic score, but not with mean polygenic score. These studies lead us to expect the presence of genetic heterogeneities in response to trauma by a variance polygenic score.

### 1.2. 9/11 as trauma

9/11 was a series of terrorist attacks by al-Qaeda on the morning of September 11, 2001. Aircrafts were crashed into the World Trade Center in Manhattan, New York; the Pentagon in Arlington County, Virginia; and Stonycreek Township in Shanksville, Pennsylvania. Approximately 3,000 people died and more than 6,000 people were injured. Adult New Yorkers who repeatedly saw victims falling from the World Trade Center reported a higher prevalence of posttraumatic stress disorder and depressive symptoms than other New York residents (Ahern et al., 2002). Victims of 9/11 were not restricted to individuals physically presented in the targeted areas. The repeated exposure to graphic media images of 9/11 predicted an increase of posttraumatic stress disorder (Ahern et al., 2002; Silver et al., 2013).

Previous studies provide empirical evidence for different responses to 9/11 among New Yorkers. For example, a longitudinal study over 12 years found four different trajectories of post-traumatic stress disorder among the World Trade Center disaster responders: 76.1% of resilience, 7.5% of recovery, 12.1% of delayed, and 4.4% of chronic trajectories (Feder et al., 2016). Further, distal or proximal exposure played an important role for a predictor of the response to trauma. 55.6% of individuals exposed to 9/11 in New York showed a resilient profile, whereas the proportion of resilient individuals dropped by one-third when restricted to physically injured individuals (Bonanno et al., 2006). In addition, peritraumatic stress and childhood interpersonal trauma are also positively associated with posttraumatic stress after 9/11 (Simeon et al., 2008). Responses to 9/11 were also different by socio-demographic characteristics. Males and those with less than a high school education showed a higher likelihood of limited psychological disturbance than females and more educated individuals (Bonanno et al., 2005, 2007).

### 1.3. Genotype and policy intervention

“Should we seek to identify the most susceptible children and disproportionately target them when it comes to investing scarce intervention and service dollars? I believe the answer is yes” (Belsky, 2014).

The availability of genetic data in social scientific datasets makes scholars question whether genotypes can be used to target susceptible individuals to increase the efficiency of policy interventions. Despite the growing interests in using genotypes to maximize benefits of policy intervention, there are remaining challenges that scholars should resolve to suggest such genetic screening. Boardman and Fletcher (Boardman & Fletcher, 2015) encouraged scholars to consider the relatively small magnitude in many measured gene-environment interactions. One important question is whether the exposed population can be divided into those with large responses to trauma and those with, essentially, no responses; in these cases, the benefits to differential treatment based on genotype may be largest. Alternatively, it could be the case that there are both differences in responses to trauma, but that everyone exposed faces substantial reductions in mental health outcomes. In this case, the advantages of differential treatment may be lower and the full exposed population may benefit from access to treatment. Overall, researchers need to resolve these open questions in order to both gain understanding of differences in responses to trauma and in considering the returns to targeting treatment regimens based on genotype.

### 1.4. The current study

The diatheses-stress model leads us to test for genetic heterogeneities in response to trauma. Nonetheless, no studies have used polygenic score measurements to examine the genetic heterogeneities in immediate response to trauma for young adults. To fill this gap in the existing literature, we test the following hypothesis by using an established quasi-experimental design:

**Hypothesis 1**. Young adults who carry a higher genetic risk of psychological problems are more likely to suffer from psychological disturbance immediately after 9/11 than their counterparts who carry a lower genetic risk of psychological problems.

Further, we also test the following hypothesis in consideration of a recent methodological advancement of variance polygenic score:

**Hypothesis 2**. Young adults who carry a higher genetic plasticity of psychological problems to environmental shocks are more likely to suffer from psychological disturbance immediately after 9/11 than their counterparts who carry a lower genetic plasticity.

By testing these hypotheses, we also aim to provide some assessment of whether benefits of genetic screening to identify susceptible individuals after trauma are plausible.

## 2. Data and methods

### 2.1. Data

We used data from the National Longitudinal Study of Adolescent to Adult Health (Add Health) (Harris et al., 2009). Add Health is a nationally representative longitudinal study of adolescents of 7th to 12th grade students in 1994 and 1995 in the U.S. The Wave III is a follow-up survey interviewed from August 2001 to April 2002, when original respondents were between the ages of 18 and 26.15,170 out of 20,745 Wave I respondents completed interviews in the Wave III. Of the completed respondents in the Wave III, we excluded those who were non-European genetic ancestry (n = 10,318) to increase the ancestral homogeneity of our sample (differences in demographic characteristics and phenotypes by ancestry groups are provided in Appendix A). Additionally, those who lack genetic markers (n = 103) and information of the dependent variable (n = 23) were also excluded. Our total analytical sample was 4,726. We also used the Wave I to construct some control variables.

### 2.2. Identification strategy

To investigate genetic heterogeneities in response to trauma, we used a sharp regression discontinuity design (RDD) (Thistlewaite & Campbell, 1960). An RDD requires two key variables: (1) a binary treatment variable and (2) a running variable. A binary treatment variable indicates whether respondents are classified in a treatment group or a control group. A running variable is a determinant of treatment assignment. The RDD is an appropriate identification strategy to estimate a local average treatment effect at the exclusive and exhaustive cut-point by comparing neighbors around the cut-point. The RDD is well suited to our purposes because we are interested in the *immediate* response to trauma. The RDD assumes no other treatment and exchangeability around cut-points, which implies respondents cannot sort around the threshold.

### 2.3. Variables

#### 2.3.1. Dependent variable

We used a set of questions from a Center for Epidemiology Studies Depression (CES-D) screener (Radloff, 1977) asking respondents how often each of the following things was true during the past seven days: (1) you were bothered by things that usually do not bother you; (2) you could not shake off the blues, even with help from your family and friends; (3) you felt that you were just as good as other people; (4) you had trouble keeping your mind on what you were doing; (5) you were depressed; (6) you were too tired to do things; (7) you enjoyed life; (8) you were sad; and (9) you felt that people disliked you. The answer for each question ranges from 0 (never) to 3 (most/all the time). A depression scale was constructed by summing these items after reverse-coding (3) and (7) to synthesize with other variables. The CES-D screener ranged from 0 (least depressed) to 27 (most depressed) with Cronbach’s alpha value of 0.808.

#### 2.3.2. Polygenic scores

We employed two polygenic scores (also known as polygenic risk scores) as genetic measurements. A polygenic score is created using the cumulative effects of segregating loci of small effect to phenotypes (Conley, 2016). A polygenic score is constructed for prediction from Genome-Wide Association Studies (GWAS) (Young et al., 2019) and its predictive power can sometimes be substantially stronger than other genetic measures (Kong et al., 2018). Further, a polygenic score is fixed at birth. These are important features of the polygenic score because it presents the possibility that clinicians may predict susceptible individuals before trauma occurs.

Our primary genetic measurements were the mean and variance depression polygenic scores. We used the mean depression polygenic score provided by Add Health. Following the recommendation of Add Health (Okbay et al., 2018), we used the mean depression polygenic score constructed based on findings of multi-trait analysis of genome-wide association (MTAG) (Turley et al., 2018). Because there is no publicly available variance depression polygenic score in Add Health, we constructed a variance depression polygenic score using UK Biobank (for methodological details, see Appendix B). In order to first test for broad heterogeneities in response to 9/11 between those with high and low depression polygenic scores, we used the median of the depression polygenic scores as a threshold to define a low (≤50 percentile) and high (>50 percentile) depression polygenic score groups. Additionally, we also assessed genetic heterogeneities around the mean of the depression polygenic scores by treating the standardized depression polygenic scores as continuous measures.

As a validity check for the mean depression polygenic score, we evaluated the association between mean depression polygenic score and the CES-D scores after accounting for first 10 principal components to account for population structure-related confounding effects (Price et al., 2006) in our analytical sample. We employed an ordinary regression to evaluate this association. We found that the CES-D score in Wave III for a high mean depression polygenic score group is 0.53 points higher than that for those with a low mean depression polygenic score (p < 0.001). Furthermore, the dichotomous measure of mean depression polygenic score explains 0.7% of the variance in the CES-D score. These results are consistent with our expectation that a higher mean depression polygenic score is associated with higher depressive symptoms.

#### 2.3.3. Exposure to trauma

We used exposure to 9/11 as our measure of trauma. Data of Add Health Wave III were collected over 2001 and 2002, which overlapped with 9/11. The binary treatment variable was constructed from the interview dates. Individuals interviewed after 9/11 were classified into the treatment group (coded as 1), and those interviewed prior to 9/11 were classified into the control group (coded as 0). All respondents interviewed on September 11, 2001, were classified into the treatment group because the terrorist attacks occurred early on the morning of September 11. We also used the interview date as a running variable and centered at September 11, 2001. Because 9/11 consisted of a series of unexpected exogeneous traumatic events, we assumed the unidirectional causation (i.e., trauma causes depressiveness).

#### 2.3.4. Control variables

Adding control variables within an RDD setting was motivated to improve precision of the treatment effects (Imbens & Lemieux, 2008) and account for potential source of effect heterogeneities. Control variables included the CES-D score in Wave I, age, sex, baseline personality (neuroticism, extraversion, and conscientiousness based on the findings of Young and Beaujean (2011)), state of residence, family income during high school, mother’s educational attainment, and the score of the Pea-body Picture Vocabulary Test (PVT). We selected these control variables based on social, environmental, demographic predictors of response to trauma (Bonanno et al., 2011) and a previous paper with the same setting (Fletcher, 2018). For instance, controlling the state of residence accounts for the potential effect heterogeneity by geographical proximity. In addition, we also incorporated the first 10 principal components as covariates to account for population structure-related confounding effects (Price et al., 2006). Further, we also included standardized variance polygenic score in the analyses for the mean polygenic score and standardized mean polygenic score in the analyses for the variance polygenic score.

Some responses to the questions used to construct these control variables were missing, with a missing rate of 0.2% (CES-D score in Wave I) to 32% (baseline extraversion). To avoid losing respondents with missing data, we used multiple imputation via chained equations to impute missing values of these control variables (Royston, 2004). Ordered logistic regression and predictive mean matching were used for imputation models to impute categorical variables and continuous variables. We created 20 multiply imputed datasets, the number of datasets with minimum statistical power falloff (Graham et al., 2007).

### 2.4. Models

#### 2.4.1. Analytical strategy

The choice of bandwidths is a major issue in the RDD and shapes the precision and bias of estimates. We used multiple arbitrarily selected bandwidths (Dunning, 2012) to confirm the credibility of the discontinuity. We selected bandwidths within 10 days (September 1 to September 21) (Model 1), 20 days (August 22 to October 1) (Model 2), 30 days (August 12 to October 11) (Model 3), 40 days (August 2 to October 21) (Model 4), and 50 days (July 23 to October 31) (Model 5) of 9/11. Additionally, we also estimated the RDD with the entire sample (Model 6). Because our primary interest is the discontinuity at the cut-point, we used a triangular kernel weight to maximize the weight at the cut-point and decline systematically as observations go farther from the cut-point. Although the participants in the Add Health were originally clustered within schools, we used heteroskedasticity-robust standard errors, rather than cluster-robust standard errors, across the model because participants had left schools within which they were clustered at the time of survey.

We used the interview date as a running variable and set September 11, 2001, as the cut-point. Because the interview date is an exclusive and exhaustive single determinant for treatment assignment, we employed a sharp RDD. The estimated equation is as follows:

$$Y_{i}=\alpha +\beta_{1}T_{i}+\beta_{2}r_{i}+\beta_{3}\left(T_{i}*r_{i}\right)+\beta_{4}\chi_{i}+\varepsilon_{i}$$

where *T*_*i*_ indicates treatment assignment, *r*_*i*_ indicates interview dates, and ***χ***_***i***_ indicates a set of covariates except the first 10 principal components. *β*_*1*_, which is our interest, indicates the local average treatment effect of 9/11 on the CES-D score. (*β*_*1*_ + *β*_*3*_) denotes the slope of interview date after 9/11. We employed a linear equation because an appropriately chosen bandwidth adjusts the accuracy of approximation relative to higher-order polynomial equations (Cattaneo et al., 2019)and the higher-order polynomials induce unattractive weights, sensitivity to the order of the regression equation, and misleading confidence intervals (Gelman & Imbens, 2019). Then, we developed the regression equation to observe genetic heterogeneities in response to trauma:

$$Y_{i}¥=\alpha +\beta_{1}T_{i}+\beta_{2}r_{i}+\beta_{3}G_{i}+\beta_{4}\left(T_{i}*r_{i}\right)+\beta_{5}\left(T_{i}*G_{i}\right)+\beta_{6}\left(r_{i}*G_{i}\right)+\beta_{7}\left(T_{i}*r_{i}*G_{i}\right)+\beta_{8}PC_{i}+\beta_{9}\chi_{i}+\varepsilon_{i}$$

where *G*_*i*_, ***PC***_***i***_, ***χ***_***i***_ indicate the depression polygenic score, a vector of the first 10 principal components and the other covariates. *β*_*1*_ and *β*_*5*_, which are our primary interest, denote the local average treatment effect of the September 11 attacks on the CES-D score and its difference by the depression polygenic score. To allow different slopes for the interview date by polygenic scores, we added an interaction between the running variable and the depression polygenic score and a triple interaction between the dummy variable of treatment, the running variable, and the polygenic score. *β*_*7*_ indicates how the trajectories after the September 11 attacks differ by the depression polygenic score. The results of this model indicate whether genetic heterogeneities in the immediate response to trauma exist among young adults.

#### 2.4.2. Sensitivity analysis

One issue with estimating longitudinal patterns of depression is the seasonal variation in depressive symptoms (Magnusson & Boivin, 2003). The RDD addresses this issue by the inclusion of interview dates in the regression equation; however, this model specification assumes that interview date is linearly associated with depression. Indeed, preliminary analyses showed that a regression equation of a quadratic interview date term on CES-D in Wave III is fitted better than a linear regression specification for some cases. Despite the concerns of using a higher-order polynomial regression equation for RDD, we assessed the sensitivity of our results to the assumption of the linear association between the interview date and the CES-D score. Following the methodological recommendation to estimate a discontinuity with local linear, quadratic polynomial or smooth functions (Gelman & Imbens, 2019), we conducted sensitivity analyses with an assumption that the association between the interview date and the CES-D score is quadratic (see Appendix C for the methodological details). In addition, because our dependent variable is highly skewed, with a range of 0–25 and a mean of 4, we also assessed the sensitivity of our results to the skewness of the dependent variable. Following the previous studies that accounted for the skewness of the CES-D score (Cole et al., 2000; Dean et al., 1992), we conducted sensitivity analyses with natural log of the CES-D score and square-root of the CES-D score. When taking natural log of the CES-D score, we added 1 to the raw CES-D score because natural log of 0 cannot be defined. The results of sensitivity analyses demonstrated that results were generally similar to the results presented in the following section (see Appendix D for the results of sensitivity analyses).

### 2.5. Validity checks

A major disadvantage of the RDD is the necessity of larger sample sizes than experimental research (Schochet, 2009). To evaluate whether the RDD in the current study suffers from low statistical power, we calculated the statistical power for the treatment effect by using RDD power calculator (see Appendix E for results). Because the RDD power calculator did not allow us to estimate the statistical power for the interaction term, we focused on the power for the treatment effect. Our results showed that the statistical power for the treatment effect is around 0.61 to 0.88. One exception is the power in the full model, which is much lower than the power in the other models. Overall, although the power for the treatment effect did not reach the required statistical power for experimental studies (i.e., 1 – β ≥ 0.8) in most cases, using an established causal inference technique to identify the causal effect of trauma on mental health is a unique contribution to the gene-environment studies. Furthermore, a recent study proposes the importance of underpowered observational causal studies to obtain the precise pooled effect estimate in a meta-analysis (Hernán, 2021). Therefore, the power issue in our analysis is a limitation which increases the likelihood that the observed significant results may be false positive, but it does not eliminate the value of the analysis.

**Fig. 2.**
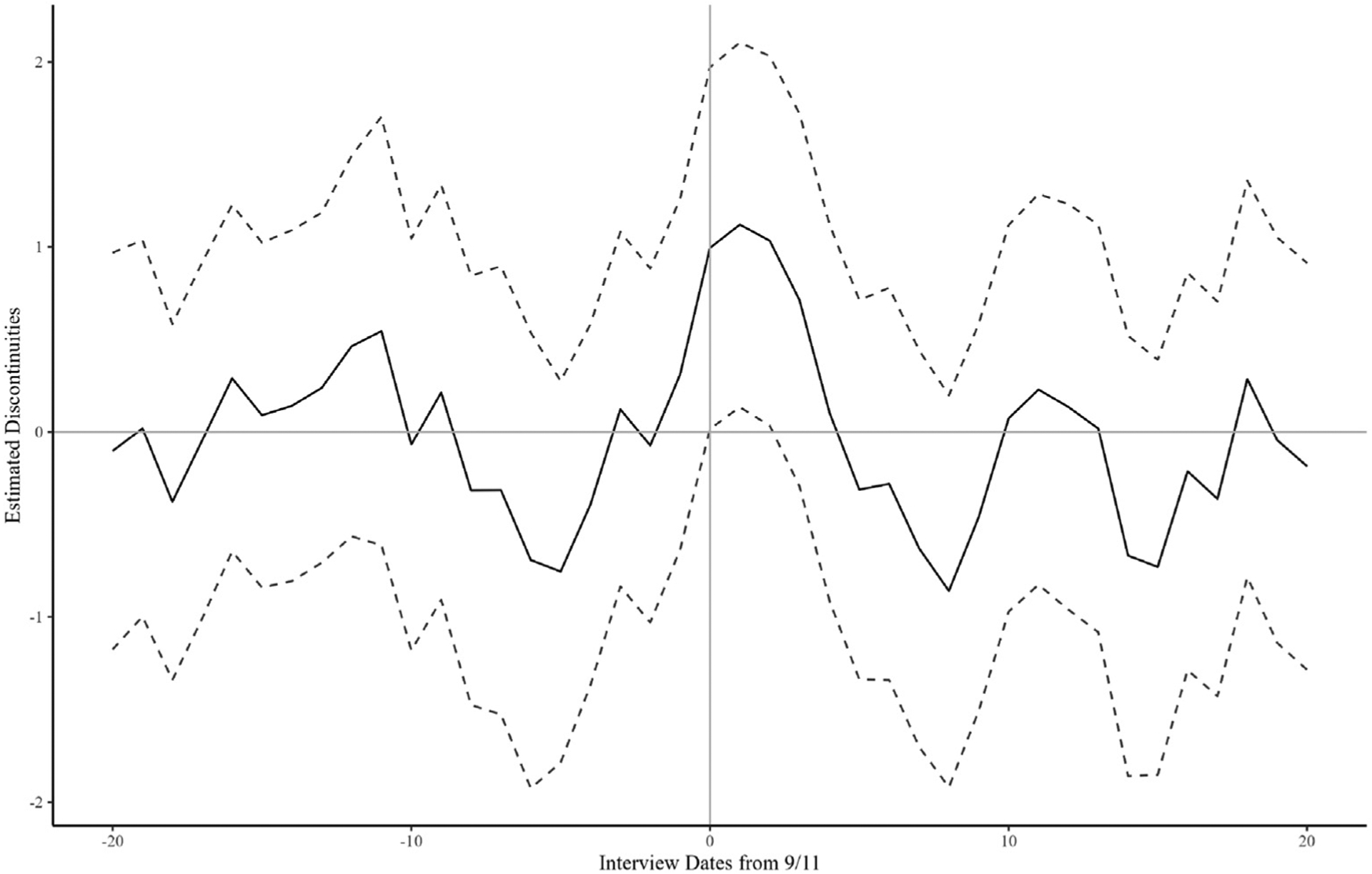
Estimated discontinuities with the cut-off points other than September 11. Note: the solid line shows the point estimators of the discontinuity, and the dashed line shows 95% confidence interval. We estimated discontinuities with the cut-off points from August 22 (equivalent to −20 on x-axis) to October 1 (equivalent to 20 on x-axis) without covariates.

Furthermore, we also conducted two additional validity checks to test the RDD assumptions: (1) no treatment other than 9/11 and (2) exchangeability around cut-points which implies respondents could not sort around the threshold. First, we conducted placebo regressions for the CES-D score to test the significance of the observed discontinuity at other interview dates (August 22 to October 1) with bandwidths of 20 days prior to and after the cut-point. Fig. 2 presents the discontinuities (the solid line) and their 95% confidence intervals (the dashed lines). Fig. 2 shows the positive discontinuities around 9/11 are highest. Additionally, the positive discontinuities around 9/11 are the only discontinuities that remain at the 5% significant level for several days. Therefore, the results of the placebo tests did not show evidence to question the validity of the RDD.

The RDD also assumes that the treatment and control groups have similar characteristics. To test this exchangeability assumption, we estimated the RDD with a dependent variable of CES-D score and 19 items of the CES-D score in Wave I, neuroticism, extraversion, conscientiousness, age, sex, state of residence where at least 40 respondents in our analytical sample lived within the bandwidths of 50 days, family income, mother’s educational attainment, PVT score, the first 10 principal components, and mean and variance depression polygenic scores with bandwidths of 50 days. Out of 64 tests, the discontinuities of the proportion of living in state 27 (β = −0.04, p < 0.05), the proportion of mothers with some college degree (β = −0.09, p < 0.05), family income (β = 14.37, p < 0.001), and principal component 9 (β = −0.00, p < 0.05) reached a conventional 5% significance level. Most results showed no significant discontinuities and the effect sizes relative to their standard deviation are not as large as that of the CES-D in Wave III. However, the number of significant discontinuities was slightly larger than would be expected by chance. This may be due to postponements of an interview among those living close to the stricken areas. Nonetheless, the Add Health anonymizes the place of residence and does not provide information on interview postponements; therefore, we cannot empirically evaluate that speculation. Instead, we added state of residence as a control variable to account for this possibility. After controlling state of residence, the discontinuity of the proportion of mothers with some college degree was no longer statistically significant. Overall, we found no evidence to question the validity of the RDD in the balance check. Additionally, the non-significant discontinuities of mean and variance depression polygenic scores demonstrate the validity of gene-environment analysis, which assumes no gene-environment correlation.

## 3. Results

### 3.1. Descriptive statistics

Table 1 presents descriptive statistics for all variables in our analyses separately by the control and treatment groups except the state-level fixed effects. Statistical tests show significant differences between before and after 9/11 for the interview date and age. This is because all respondents in the treatment group were interviewed after the control group. Relatedly, the respondents in the treatment group were slightly older due to the difference in interview date. Among covariates, we do not observe statistically significant differences between before and after 9/11 except the proportion of sex and the principal component 2. Because we found no evidence to question the validity of the RDD, the difference in the proportion of sex between before and after 9/11 does not reduce the validity of our identification strategy. Further, the differences in the mean and variance depression polygenic score between the treatment and control group are very small and not statistically significant. This is additional empirical evidence for the validity of the gene-environment interaction analysis, which assumes no gene-environment correlation.

### 3.2. 9/11 and depressive symptoms

**Fig. 3.**
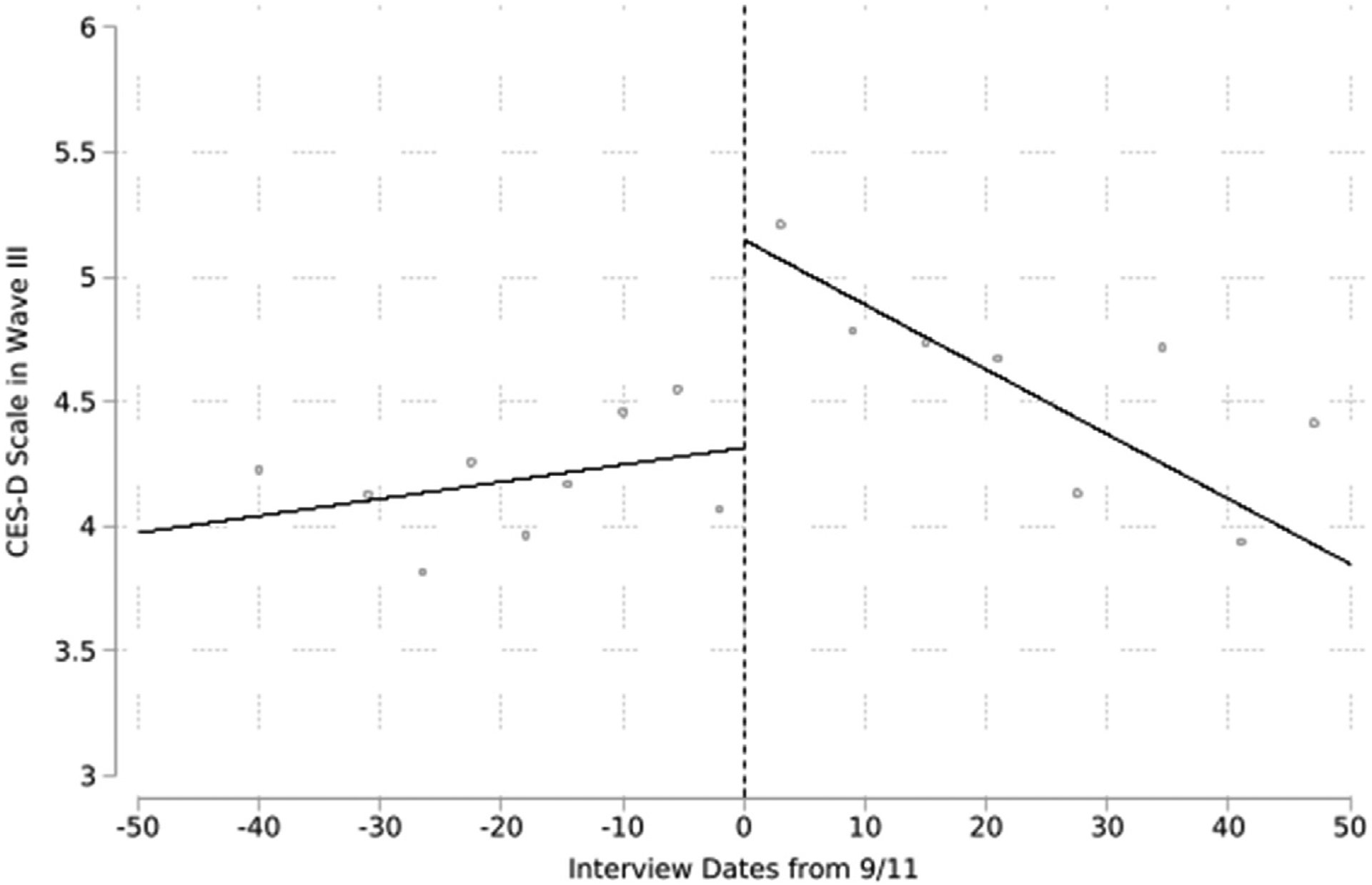
The visualized results of regression discontinuity after the September 11 attacks. Notes: The analytical sample is restricted to participants interviewed 50 days before or after 9/11.Observations are binned by using the quantile-spaced method. Each bin contains approximately 117 observations for the control group and 159 observations for the treatment group. The number of observations per bin may vary. No control variables are adjusted.

The visualized output of the RDD with bandwidths of 50 days prior to and after 9/11 is presented in Fig. 3. We can visually confirm the discontinuity at the cut-off point—clearly demonstrating the *immediate* increase of the CES-D score after 9/11. The slope of interview dates on the CES-D score prior to 9/11 is positive, whereas the CES-D score shows negative association with the interview dates after 9/11. Results are consistent in Table 2. The discontinuities at 9/11 are statistically significant and substantially meaningful, reaching over one-fifth of a standard deviation in the CES-D score among those interviewed before 9/11 (s.d. = 3.87) in Models 1 to 5. Despite the relatively small effect size to the other models, Model 6 also shows the statistically significant discontinuity. The coefficients of interview dates are not statistically significant before 9/11 across the models; however, the association between the CES-D score and interview dates becomes negative after 9/11. For instance, an auxiliary test tells us that interview dates are negatively and significantly associated with the CES-D score in Model 6 (β = −0.006, p < 0.001).

### 3.3. Genetic heterogeneity in Response after 9/11

**Fig. 4.**
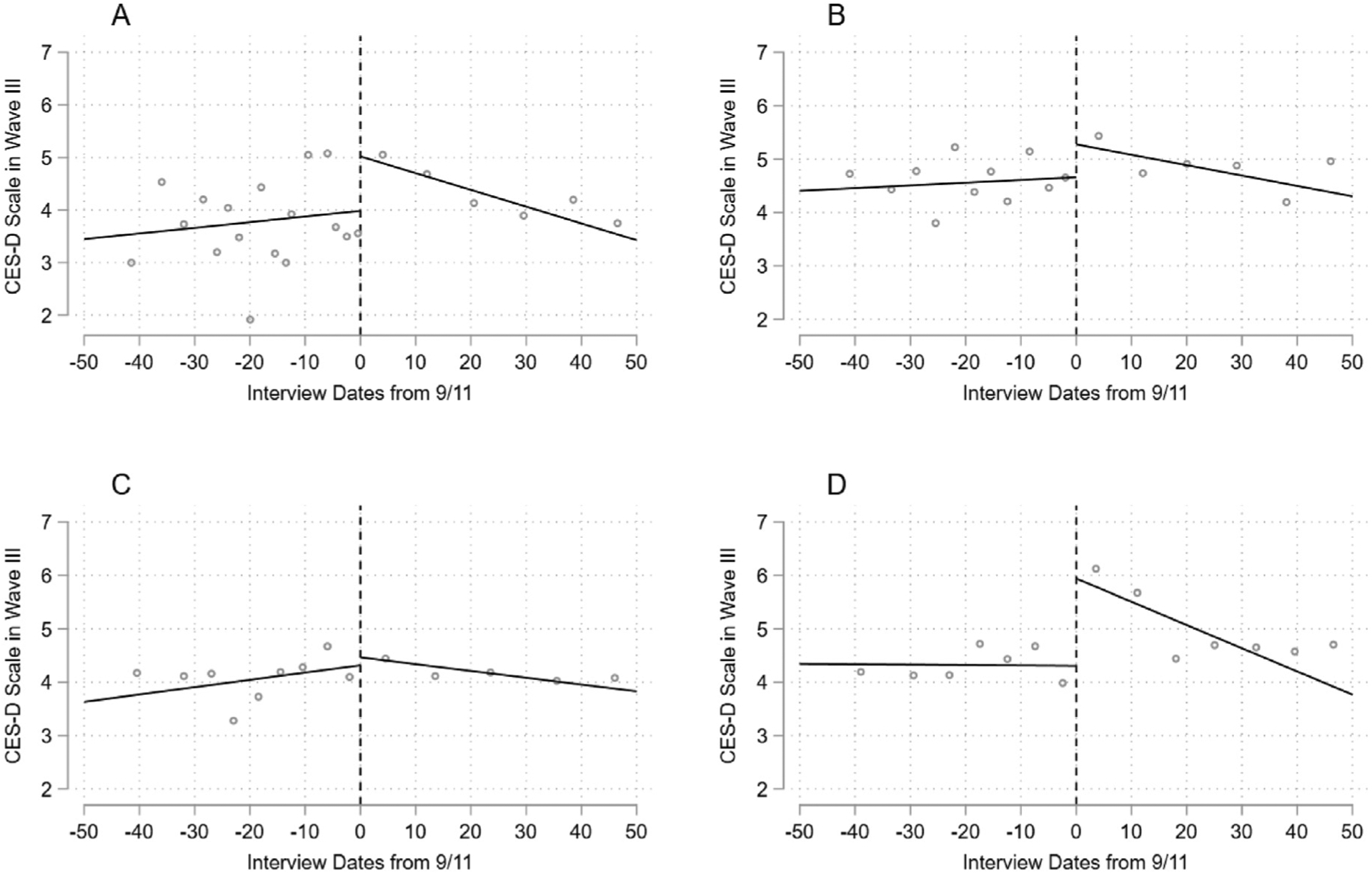
The visualized results of regression discontinuity after the September 11 attacks, by standardized mean and variance depression polygenic score groups. Notes: Panel A and B present the RDD by mean depression polygenic score and Panel C and D present the RDD by variance depression polygenic score. Panel A and C show the results for the low polygenic score group, whereas Panel B and D show the results for the high polygenic score group. These plots were estimated separately. The analytical sample is restricted to participants interviewed 50 days before or after 9/11. Observations are binned by using the quantile-spaced method. In Panel A, each bin contains approximately 30 observations for the control group and 130 observations for the treatment group. In Panel B, each bin contains approximately 49 observations for the control group and 104 observations for the treatment group. In Panel C, each bin contains approximately 58 observations for the control group and 127 observations for the treatment group. In Panel D, each bin contains approximately 75 observations for the control group and 90 observations for the treatment group. The number of observations per bin may vary. No control variables are adjusted.

Fig. 4 Panel A and B visualized the RDD with bandwidths of 50 days prior to and after 9/11 by the mean depression polygenic score (Panel A for those with low mean depression polygenic score and B for those with high mean depression polygenic score). These figures demonstrate that those with high mean depression polygenic score tend to report higher CES-D score than those with low mean depression polygenic score; however, there is only small difference in the discontinuities by these two groups. Results of the RDD by the mean depression polygenic score are presented in Table 3. The results in Model 5 show 1.21 points increase in CES-D score associated with 9/11 for those classified in the low mean depression polygenic score group. This result implies that people experienced an increase of CES-D score after trauma regardless the mean depression polygenic score groups. Furthermore, the mean depression polygenic score does not moderate the effect of 9/11 on CES-D score. On average, the effect of 9/11 on CES-D score for the high mean depression polygenic score group is 0.60 points smaller than corresponding effect for the low mean depression polygenic score group in Model 5, but this difference is not statistically significant. We also examined the genetic heterogeneities by treating the depression mean polygenic scores as a continuous measure (see Appendix F for the results with a continuous mean depression polygenic score). With the bandwidths of 50 days, results show that very small genetic heterogeneities in an increase of the CES-D score immediately after trauma (β = 0.028, p = 0.922). Additionally, our estimates of interactions between the polygenic score, treatment, and the time-since-9/11 suggest no evidence that the pace of recovery trajectory differs by the depression polygenic score.

We then turned to the RDD with a variance depression polygenic score. Fig. 4 Panel C and D visualized the RDD with bandwidths of 50 days prior to and after 9/11 by the variance depression polygenic score group (Panel C for those with low variance depression polygenic score and D for those with high variance depression polygenic score). Unlike the results for the mean depression polygenic score, the effect of 9/11 is larger for those who carry a high variance depression polygenic score than the corresponding effect for those with a low variance depression polygenic score. These are consistent with the results presented in Table 4. Whereas those with a low variance depression polygenic score do not experience a significant increase of CES-D score after 9/11, there is a substantial increase in CES-D score after 9/11 for those with high variance depression polygenic score. Further, the coefficient for the interaction between the treatment and the continuous measure of the standardized variance depression polygenic score is 10 times larger (β = 0.275) than the corresponding coefficient for the standardized mean depression polygenic score with the bandwidths of 50 days, suggesting the necessity to use the variance depression polygenic score to observe the potential genetic heterogeneities in response to 9/11 (see Appendix F for the results for a continuous variance depression polygenic score). Nonetheless, it should be noted that this genetic heterogeneity is not statistically significant (p = 0.362); therefore, there is no significant interaction around the mean of the depression variance polygenic score. Additionally, the analysis with the standardized variance depression polygenic score suggests that those who were classified into the low polygenic score group still experienced an increase of CES-D score after 9.11. Specifically, for those with standardized variance polygenic score of −0.5, we expect to see 0.689 points increase in CES-D score at the 10% significance level. Although we virtually observe a steeper decline of CES-D score for the high variance depression polygenic score group than for the low variance depression polygenic score group in Fig. 4, there are no significant genetic heterogeneities in the pace of recovery trajectory with the variance depression polygenic score.

## 4. Discussion

Our goal in this study was to evaluate genetic heterogeneities in the immediate response to trauma with a quasi-experimental design. Our first finding, replicating other works, is an increase of the CES-D score immediately after 9/11. We also found that the CES-D score was not associated with the interview dates prior to 9/11 but negatively associated with the interview dates after 9/11—following a recovery trajectory. These results match theoretical predictions because the majority of the sample are expected to recover to the normal level of depressive symptoms even if they experience an increase of depressive symptoms immediately after trauma (Bonanno, 2004; Bonanno et al., 2011).

We considered genetic heterogeneities in response to trauma by using two depression polygenic scores. Using the mean depression polygenic score, there were no detectable genetic heterogeneities in response to trauma. This is likely because the mean depression polygenic score captures the level of psychological distress, but not the plasticity of mental health to environmental exposures. Nevertheless, our findings are not consistent with the diathesis-stress model (Monroe & Simons, 1991) and the previous findings with an experience of spousal death among older American residents (Domingue et al., 2017). Subsequent research is required to investigate in which case we observe the genetic heterogeneities in psychological disturbance after trauma experiences with the mean depression polygenic score.

By contrast, individuals with a heightened genetic plasticity/sensitivity of mental health to environmental exposures showed a greater vulnerability immediately after trauma than those with a lower genetic plasticity. This finding is particularly important in light of the emerging interest in genetic plasticity in gene-environment research. Consistent with previous research (Johnson et al., 2021; Schmitz et al., 2021), our results also suggest that the variance polygenic scores may shed light on gene-environmental interactions which might be unobservable with mean polygenic scores. Furthermore, given the empirical evidence for the association between short-term increase of depressive symptom and long-term poor health outcomes (Domingue et al., 2020), the presence of genetic heterogeneities in response to trauma implies that those with a heightened genetic plasticity may be more likely to suffer from long-term unfavorable health outcomes after a trauma experience than those with a lower genetic plasticity. Additionally, these results also suggest that the response to trauma may be partially determined by genetic factors.

Despite the presence of suggestive visual results for differences in the pace of recovery by the variance depression polygenic score, we did not find statistically significant differences in the pace of recovery by the mean or variance depression polygenic scores. Although bereavements cannot be equated with exposure to 9/11, this is not consistent with the findings that the pace of recovery after a stressful life event is faster among older people with a high mean depression polygenic score than those with a low mean depression polygenic score (Domingue et al., 2017). This inconsistency may be due to differences in research design and the analytical sample. For instance, Domingue and colleagues (2017) used a measure in months following stressful life event, whereas we used a measure in days. Further, we focused on young adults while the analytical sample of Domingue and colleagues (2017) was older people. These differences suggest the necessity of subsequent research evaluating how and to what extent genetic heterogeneities in the pace of recovery vary by, for example, age, unit of the measures of pace, degree/type of trauma, and expected and unexpected trauma experiences.

Our results also provide important implications for policy interventions. We confirmed a larger increase of depressive symptoms after 9/11 among those with a high variance depression polygenic score. This implies the possibility of policy interventions to improve the pace of recovery to achieve the normal level of depressive symptoms for those who experienced a larger increase of depressive symptoms after trauma. Nevertheless, given that the suggested cut-off score for significant depressive symptoms in the 10-item CES-D score is 8 or above (Andresen et al., 1994; Björgvinsson et al., 2013), the effect sizes of genetic heterogeneities in the immediate increase of depressive symptoms are too small to identify susceptible clinical patients. We, therefore, contend that the expected benefits from genetic screening to identify a susceptible group after trauma are limited. This is consistent with Boardman and Fletcher’s (Boardman & Fletcher, 2015) caution against attempting to leverage small magnitudes of gene-environment interaction in considering treatment decisions. Additionally, although we classified respondents into high and low variance depression polygenic score groups to examine broad genetic heterogeneity in response to 9/11, sizeable numbers of people might still experience an immediate increase of depressive symptoms after trauma even for those who were classified into the low polygenic score group. This suggests that clinicians may fail to intervene on those who experienced an increase of depressiveness after trauma if they target even when they use the variance depression polygenic score. These results imply that failure to select an appropriate polygenic score and to set a proper threshold of a polygenic score for policy interventions would lead policy makers to miss victims in need of public support.

Our results provide an empirical basis for genetic heterogeneities in the immediate response to trauma, but the study is limited in several ways. First, the nature of current social genomics research precludes the inclusion of non-European ancestry respondents. Because the accuracy of prediction of polygenic scores relies on training GWAS sample sets (Young et al., 2019) and most GWAS sample sets are biased towards individuals of European ancestry (Martin et al., 2019), our analytical sample was restricted to European ancestry. The exclusion of individuals of non-European ancestry implies that our analytical sample is not a representative sample of Add Health participants and, more broadly, young adults. In addition, we caution that the focus on European ancestry individuals may exacerbate existing health disparities given that social genomics research does not ubiquitously contribute to better understandings in gene-environment interaction across the genetic ancestry groups (Martin et al., 2019). The expansion of GWAS sample sets will enable researchers to include individuals of non-European ancestry, increase the sample size, and extend our research question to, for example, how and to what extent genetic heterogeneities in response to trauma differ between individuals of European ancestry and non-European ancestry.

Second, the observed genetic heterogeneities in response to 9/11 may not be due to genetic differences. Because polygenic scores are constructed for prediction from GWAS, polygenic scores for socially determined outcomes may reflect not only genetic differences but also socio-environmental differences (Morris et al., 2020). Furthermore, the fact that one genotype that is associated with one trait is also associated with many other traits suggests the polygenic score to predict one trait also predicts other traits (also known as pleiotropy). This implies that the variance depression polygenic score may also be associated with potential determinants of reaction to 9/11 and the genetic heterogeneity in response to 9/11 may reflect the heterogeneity caused by non-genetic factors. In this context, the lack of correlation between the variance depression polygenic score and the treatment assignment is one important strength of the current study; however, we cannot infer the underlying mechanisms for the observed genetic heterogeneities.

Third, there may be a measurement error in the polygenic scores. Indeed, a prior MTAG study suggested the large amount of measurement error in the mean polygenic score (Turley et al., 2018). This large amount of measurement error in the polygenic score would attenuate the estimated coefficients of the interaction of the treatment and the mean depression polygenic score if it is a classical measurement error (see Appendix G for the potential impact of the measurement error on our results). Therefore, we caution that the genetic heterogeneity in response to 9/11 by the mean depression polygenic score may exist if there is the large amount of measurement error in our mean depression polygenic score.

Finally, given that the majority of the sample does not develop chronic psychological problems, our approach precludes us from identifying the differences in genetic profiles among resilient, recovery, delayed distress, and chronic distress trajectories. How do genetic profiles among those who experienced the delayed or chronic distress trajectories differ from those who experienced the resilient or recovery trajectories? Studies to address such question would advance the discussion of policy interventions by genotypes for a chronic mental disorder after trauma.

Despite these limitations, our study makes valuable contributions to empirical evidence of genetic heterogeneities in response to trauma. We were able to document genetic heterogeneities in response to 9/11 by using a variance depression polygenic score, an emerging genetic marker for gene-environment research. Of particular importance for the current debate in policy interventions is evidence for the limited benefits of genetic screening to efficiently intervene susceptible individuals. We also documented the possibility that policy makers may fail to intervene with victims in need of public support if they rely on genotypes to target susceptible individuals. Although we cannot argue that policy makers should depend on genetic markers to target susceptible individuals, these novel insights provide an additional heterogeneity contributing to the inequality of health. Subsequent extensions of this research may investigate differences in genetic heterogeneities by non-European ancestry groups with more diverse GWAS training datasets, use a larger sample with a stronger statistical power to detect more precise genetic heterogeneities in response to trauma, and examine genetic profiles among the four prototypes of the trajectory of response to trauma. A growing availability of genetic data in nationally representative datasets has been stimulating scholarly interests among social scientists and makes it possible to not only control genetic markers but also develop a novel dimension of heterogeneity. Revisiting accumulated wisdoms in social sciences with genetic data and investigating interactive associations between genotypes and environmental factors are important tasks for social scientists to deepen the understandings of social phenomena.

## Supplementary Material

1

## Figures and Tables

**Table 1:** Descriptive statistics.

VARIABLES	Before 9/11		Post 9/11		Number of
	Mean/Proportion	S.D.	Mean/Proportion	S.D.	Imputed Values
CES-D score in Wave III	4.19	3.87	4.26	3.91	0
Interview date	−24.36	28.74	84.88***	59.41	0
Mean depression polygenic score					0
Low	0.52	–	0.51	–	
High	0.48	–	0.49	–	
Variance depression polygenic score					0
Low	0.50	–	0.50	–	
High	0.50	–	0.50	–	
Age	21.51	1.75	21.91***	1.76	0
Sex					0
Male	0.42	–	0.47**	–	
Female	0.58	–	0.53	–	
Mother’s educational attainment					746
Below high school	0.09	–	0.10	–	
High school	0.33	–	0.33	–	
Some college	0.33	–	0.31	–	
College graduates	0.25	–	0.25	–	
PVT score in Wave I	105.40	11.61	105.07	11.73	207
Family income	50.75	45.25	52.22	53.31	831
CES-D score in Wave I	10.88	7.49	10.41	7.36	9
Neuroticism	11.54	3.60	11.41	3.51	14
Extraversion	7.07	2.74	7.07	2.78	1,489
Conscientious	9.05	2.65	9.01	2.53	44
Principal component 1	0.00	0.01	0.00	0.01	0
Principal component 2	0.00	0.01	−0.00*	0.01	0
Principal component 3	−0.00	0.01	−0.00	0.01	0
Principal component 4	0.00	0.01	−0.00	0.02	0
Principal component 5	0.00	0.02	0.00	0.01	0
Principal component 6	−0.00	0.01	−0.00	0.01	0
Principal component 7	−0.00	0.01	−0.00	0.01	0
Principal component 8	−0.00	0.02	0.00	0.01	0
Principal component 9	−0.00	0.01	0.00	0.02	0
Principal component 10	−0.00	0.02	0.00	0.01	0
Observations	1,098		3,628		

Note: Estimated with multiply imputed datasets (n = 20).

*** p < 0.001,

** p < 0.01,

* p < 0.05,

† p < 0.1.

**Table 2:** Results of the regression discontinuity of the depressive symptom after the September 11 attacks.

VARIABLES	Model 1	Model 2	Model 3	Model 4	Model 5	Model 6
Treatment (ref: Before 9/11)	1.45* (0.64)	0.98* (0.45)	0.96* (0.39)	0.91** (0.34)	0.89** (0.32)	0.61** (0.23)
Interview date	−0.16 (0.11)	−0.00 (0.03)	0.01 (0.02)	0.01 (0.01)	0.00 (0.01)	0.00 (0.01)
Treatment × Interview date	0.18 (0.13)	−0.02 (0.04)	−0.04 (0.03)	−0.03 (0.02)	−0.02 (0.01)	−0.01 (0.01)
Constant	2.05 (3.29)	3.48 (2.29)	4.25* (1.87)	4.57** (1.65)	4.81** (1.51)	5.50*** (1.01)
Observations	563	1,134	1,639	2,027	2,318	4,726

Note: Robust standard errors in parentheses. Additional controls not shown. Estimated with multiply imputed datasets (n = 20).

*** p < 0.001,

** p < 0.01,

* p < 0.05,

† p < 0.1.

**Table 3:** Results of regression discontinuity of the depressive symptom after the September 11 attacks: interactions with the mean depression polygenic score.

VARIABLES	Model 1	Model 2	Model 3	Model 4	Model 5	Model 6
Treatment (ref: Before 9/11)	2.03* (0.80)	1.40* (0.61)	1.26* (0.53)	1.25** (0.48)	1.21** (0.45)	0.69* (0.30)
Interview date	−0.30* (0.15)	−0.03 (0.04)	0.01 (0.02)	0.00 (0.02)	0.00 (0.02)	0.00 (0.01)
Treatment × Interview date	0.39* (0.18)	0.01 (0.06)	−0.04 (0.03)	−0.04 (0.02)	−0.03 (0.02)	−0.01 (0.01)
Mean depression PGS (ref: Low)	1.99 (1.03)	0.92 (0.64)	0.53 (0.54)	0.58 (0.49)	0.58 (0.46)	0.54 (0.36)
Treatment × Mean depression PGS	−1.40 (1.20)	−0.78 (0.88)	−0.51 (0.76)	−0.62 (0.69)	−0.60 (0.64)	−0.11 (0.43)
Interview date × Mean depression PGS	0.33 (0.22)	0.06 (0.06)	−0.01 (0.03)	0.00 (0.03)	0.00 (0.02)	−0.00 (0.01)
Treatment × Interview date × Mean depression PGS	−0.44 (0.28)	−0.06 (0.09)	0.02 (0.05)	0.02 (0.04)	0.02 (0.03)	−0.00 (0.01)
Standardized variance depression PGS	0.18 (0.16)	0.22 (0.13)	0.18 (0.10)	0.14 (0.09)	0.12 (0.09)	0.17** (0.06)
Constant	0.88 (3.15)	2.80 (2.23)	3.80* (1.84)	4.14* (1.63)	4.41** (1.50)	5.11*** (1.01)
Observations	563	1,134	1,639	2,027	2,318	4,726

Note: Robust standard errors in parentheses. Additional controls not shown. Estimated with multiply imputed datasets (n = 20).

Model 1, 2, 3, 4, and 5 has bandwidths of 10 days, 20 days, 30 days, 40 days, and 50 days, respectively. Model 6 includes all observations in our analytical sample.

*** p < 0.001,

** p < 0.01,

* p < 0.05,

† p < 0.1.

**Table 4:** Results of regression discontinuity of the depressive symptom after the September 11 attacks: interactions with the variance depression polygenic score.

VARIABLES	Model 1	Model 2	Model 3	Model 4	Model 5	Model 6
Treatment (ref: Before 9/11)	1.44† (0.85)	0.26 (0.62)	0.15 (0.53)	0.18 (0.47)	0.25 (0.44)	0.23 (0.31)
Interview date	−0.29† (0.16)	0.01 (0.04)	0.01 (0.02)	0.01 (0.02)	0.00 (0.02)	0.00 (0.01)
Treatment × Interview date	0.29 (0.18)	−0.02 (0.06)	−0.02 (0.03)	−0.01 (0.02)	−0.01 (0.02)	−0.01 (0.01)
Variance depression PGS (ref: Low)	0.88 (0.97)	−0.19 (0.65)	−0.19 (0.56)	−0.13 (0.50)	−0.06 (0.47)	−0.07 (0.36)
Treatment × Variance depression PGS	0.13 (1.26)	1.59† (0.91)	1.76* (0.78)	1.60* (0.69)	1.40* (0.64)	0.79† (0.43)
Interview date × Variance depression PGS	0.27 (0.22)	−0.01 (0.06)	−0.02 (0.04)	−0.01 (0.03)	−0.00 (0.02)	−0.00 (0.01)
Treatment × Interview date × Variance depression PGS	−0.21 (0.27)	−0.02 (0.09)	−0.04 (0.05)	−0.04 (0.04)	−0.04 (0.03)	−0.00 (0.01)
Standardized mean depression PGS	0.08 (0.16)	0.10 (0.12)	0.15 (0.10)	0.17† (0.09)	0.20* (0.08)	0.21*** (0.06)
Constant	1.53 (3.26)	3.32 (2.28)	4.07* (1.87)	4.37** (1.65)	4.59** (1.52)	5.27*** (1.03)
Observations	563	1,134	1,639	2,027	2,318	4,726

Note: Robust standard errors in parentheses. Additional controls not shown. Estimated with multiply imputed datasets (n = 20).

Model 1, 2, 3, 4, and 5 has bandwidths of 10 days, 20 days, 30 days, 40 days, and 50 days, respectively. Model 6 includes all observations in our analytical sample.

*** p < 0.001,

** p < 0.01,

* p < 0.05,

† p < 0.1.

## References

[R1] Ahern J, Galea S, Resnick H, Kilpatrick D, Bucuvalas M, Gold J, & Vlahov D (2002). Television images and psychological symptoms after the September 11 terrorist attacks. Psychiatry, 65(4), 289–300. 10.1521/psyc.65.4.289.20240 12530330

[R2] Andresen EM, Malmgren JA, Carter WB, & Patrick DL (1994). Screening for depression in well older adults: Evaluation of a short form of the CES-D (Center for epidemiologic studies depression scale). American Journal of Preventive Medicine, 10(2), 77–84. 8037935

[R3] Belsky J (2014). The downside of resilience. https://www.nytimes.com/2014/11/30/opinion/sunday/the-downside-of-resilience.html. (Accessed 27 February 2021).

[R4] Björgvinsson T, Kertz SJ, Bigda-Peyton JS, McCoy KL, & Aderka IM (2013). Psychometric properties of the CES-D-10 in a psychiatric sample. Assessment, 20(4), 429–436. 10.1177/1073191113481998 23513010

[R5] Boardman JD, & Fletcher JM (2015). The promise of integrating genetics into policy analysis. Journal of Policy Analysis and Management, 34(3), 493–496. 10.1002/pam.21839

[R6] Bonanno GA (2004). Loss, trauma, and human resilience: Have we underestimated the human capacity to thrive after extremely aversive events? American Psychologist, 59(1), 20–28. 10.1037/0003-066X.59.1.20 14736317

[R7] Bonanno GA, Galea S, Bucciarelli A, & Vlahov D (2006). Psychological resilience after disaster: New York City in the aftermath of the september 11th terrorist attack. Psychological Science, 17(3), 181–186. 10.1111/j.1467-9280.2006.01682.x 16507055

[R8] Bonanno GA, Galea S, Bucciarelli A, & Vlahov D (2007). What predicts psychological resilience after disaster? The role of demographics, resources, and life stress. Journal of Consulting and Clinical Psychology, 75(5), 671–682. 10.1037/0022-006X.75.5.671 17907849

[R9] Bonanno GA, Rennicke C, & Dekel S (2005). Self-enhancement among high-exposure survivors of the september 11th terrorist attack: Resilience or social maladjustment? Journal of Personality and Social Psychology, 88(6), 984–998. 10.1037/0022-3514.88.6.984 15982117

[R10] Bonanno GA, Westphal M, & Mancini AD (2011). Resilience to loss and potential trauma. Annual Review of Clinical Psychology, 7, 511–535. 10.1146/annurev-clinpsy-032210-104526 21091190

[R11] Brewin CR, Andrews B, & Valentine JD (2000). Meta-analysis of risk factors for posttraumatic stress disorder in trauma-exposed adults. Journal of Consulting and Clinical Psychology, 68(5), 748–766. 10.1037/0022-006X.68.5.748 11068961

[R12] Burt CH, Simons RL, & Gibbons FX (2012). Racial discrimination, ethnic-racial socialization, and crime. American Sociological Review, 77(4), 648–677. 10.1177/0003122412448648 24058204 PMC3777442

[R13] Cattaneo MD, Idrobo N, & Titiunik R (2019). A practical introduction to regression discontinuity designs. Cambridge, United Kingdom: Cambridge University Press. 10.1017/9781108684606

[R14] Cole SR, Kawachi I, Maller SJ, & Berkman LF (2000). Test of item-response bias in the CES-D scale: Experience from the new haven EPESE study. Journal of Clinical Epidemiology, 53, 285–289. 10.1016/S0895-4356(99)00151-1 10760639

[R15] Conley D (2016). Socio-genomic research using genome-wide molecular data. Annual Review of Sociology, 42, 275–299. 10.1146/annurev-soc-081715-074316

[R16] Dean A, Kolody B, Wood P, & Matt GE (1992). The influence of living alone on depression in elderly persons. Journal of Aging and Health, 4, 3–18. 10.1177/089826439200400101

[R17] Domingue BW, Duncan L, Harrati A, & Belsky DW (2020). Short-term mental health sequelae of bereavement predict long-term physical health decline in older adults: U.S. Health and retirement study analysis. Journals Gerontology Series B gbaa044. 10.1093/geronb/gbaa044 PMC8200357 32246152

[R18] Domingue BW, Liu H, Okbay A, & Belsky DW (2017). Genetic heterogeneity in depressive symptoms following the death of a spouse: Polygenic score analysis of the U.S. Health and retirement study. American Journal of Psychiatry, 174(10), 963–970. 10.1176/appi.ajp.2017.16111209 28335623 PMC5610918

[R19] Duncan LE, Ratanatharathorn A, Aiello AE, Almli LM, Amstadter AB, (2018). Largest GWAS of PTSD (N=20 070) yields genetic overlap with schizophrenia and sex differences in heritability. Molecular Psychiatry, 23(3), 666–673. 10.1038/mp.2017.77 28439101 PMC5696105

[R20] Dunning T (2012). Natural experiments in the social sciences: A design-based approach. Cambridge, United Kingdom: Cambridge University Press. 10.1017/CBO9781139084444

[R21] Feder A, Mota N, Salim R, Rodriguez J, Singh R, Schaffer J, Schechter CB, Cancelmo LM, Bromet EJ, Katz CL, Reissman DB, Ozbay F, Kotov R, Crane M, Harrison DJ, Herbert R, Levin SM, Luft BJ, Moline JM, … Pietrzak RH (2016). Risk, coping and PTSD symptom trajectories in World Trade Center responders. Journal of Psychiatric Research, 82, 68–79. 10.1016/j.jpsychires.2016.07.003 27468166

[R22] Fergusson DM, Horwood LJ, & Ridder EM (2005). Partner violence and mental health outcomes in a New Zealand birth cohort. Journal of Marriage and Family, 67(5), 1103–1119. 10.1111/j.1741-3737.2005.00202.x

[R23] Fletcher J (2018). Crushing hope: Short term responses to tragedy vary by hopefulness. Social Science & Medicine, 201, 59–62. 10.1016/j.socscimed.2018.01.039 29438878 PMC6714575

[R24] Galatzer-Levy IR, Huang SH, & Bonanno GA (2018). Trajectories of resilience and dysfunction following potential trauma: A review and statistical evaluation. Clinical Psychology Review, 63, 41–55. 10.1016/j.cpr.2018.05.008 29902711

[R25] Gelernter J, Sun N, Polimanti R, Pietrzak R, Levey DF, (2019). Genome-wide association study of post-traumatic stress disorder reexperiencing symptoms in >165,000 US veterans. Nature Neuroscience, 22(9), 1394–1401. 10.1038/s41593-019-0447-7 31358989 PMC6953633

[R26] Gelman A, & Imbens G (2019). Why high-order polynomials should not be used in regression discontinuity designs. Journal of Business & Economic Statistics, 37(3), 447–456. 10.1080/07350015.2017.1366909

[R27] Graham JW, Olchowski AE, & Gilreath TD (2007). How many imputations are really needed? Some practical clarifications of multiple imputation theory. Prevention Science, 8(3), 206–213. 10.1007/s11121-007-0070-9 17549635

[R28] Harris KM, Halpern CT, Whitsel E, Hussey J, Tabor J, Entzel P, & Udry JR (2009). The national longitudinal study of adolescent to adult health: Research design. http://www.cpc.unc.edu/projects/addhealth/design. (Accessed 27 February 2021).

[R29] Hernán MA (2021). Causal analyses of existing databases: No power calculations required. Journal of Clinical Epidemiology. 10.1016/J.JCLINEPI.2021.08.028 PMC8882204 34461211

[R30] Imbens GW, & Lemieux T (2008). Regression discontinuity designs: A guide to practice. Journal of Econometrics, 142(2), 615–635. 10.1016/J.JECONOM.2007.05.001

[R31] Johnson R, Sotoudeh R, & Conley D (2021). Polygenic scores for plasticity: A new tool for studying gene-environment interplay. bioRxiv. 10.1101/2020.08.30.274530 35553650

[R32] Kim HS (2011). Consequences of parental divorce for child development. American Sociological Review, 76(3), 487–511. 10.1177/0003122411407748

[R33] Kong A, Thorleifsson G, Frigge ML, (2018). The nature of nurture: Effects of parental genotypes. Science, 359(6374), 424–428. 10.1126/science.aan6877 29371463

[R34] Levine ME, Crimmins EM, Prescott CA, Phillips D, Arpawong TE, & Lee J (2014). A polygenic risk score associated with measures of depressive symptoms among older adults. Biodemography and Social Biology, 60(2), 199–211. 10.1080/19485565.2014.952705 25343367 PMC4298361

[R35] Magnusson A, & Boivin D (2003). Seasonal affective disorder: An overview. Chronobiology International, 20(2), 189–207. 10.1081/CBI-120019310 12723880

[R36] Martin AR, Kanai M, Kamatani Y, Okada Y, Neale BM, & Daly MJ (2019). Clinical use of current polygenic risk scores may exacerbate health disparities. Nature Genetics, 51(4), 584–591. 10.1038/s41588-019-0379-x 30926966 PMC6563838

[R37] Masten AS (2001). Ordinary magic resilience processes in development. American Psychologist, 56, 227–238. 10.1037/0003-066X.56.3.227 11315249

[R38] Miao J, Lin Y, Wu Y, Zheng B, Schmitz LL, Fletcher JM, & Lu Q (2021). A quantile integral linear model to quantify genetic effects on phenotypic variability. bioRxiv. 10.1101/2021.04.14.439847 PMC9522331 36122202

[R39] Monk EP (2015). The cost of color: Skin color, discrimination, and health among African-Americans. American Journal of Sociology, 121(2), 396–444. 10.1086/682162 26594713

[R40] Monroe SM, & Simons AD (1991). Diathesis-stress theories in the context of life stress research implications for the depressive disorders. Psychological Bulletin, 110(3), 406–425. 10.1037/0033-2909.110.3.406 1758917

[R41] Morris TT, Davies NM, & Smith GD (2020). Can education be personalised using pupils’ genetic data? Elife, 9, Article e49962. 10.7554/eLife.49962 32151313 PMC7064332

[R42] Nievergelt CM, Maihofer AX, Klengel T, Atkinson EG, Chen CY, (2019). International meta-analysis of PTSD genome-wide association studies identifies sexand ancestry-specific genetic risk loci. Nature Communications, 10(1), 1–16. 10.1038/s41467-019-12576-w PMC6783435 31594949

[R43] Norris FH, Friedman MJ, Watson PJ, Byrne CM, Diaz E, & Kaniasty K (2002). 60,000 disaster victims speak: Part I. An empirical review of the empirical literature, 1981–2001 Psychiatry, 65(3), 207–239. 12405079 10.1521/psyc.65.3.207.20173

[R44] Okbay A, Turley P, Benjamin D, Visscher P, Braudt D, & Harris KM (2018). SSGAC polygenic scores (PGSs) in the national longitudinal study of adolescent to adult health (Add health). 10.17615/c6166f

[R45] Price AL, Patterson NJ, Plenge RM, Weinblatt ME, Shadick NA, & Reich D (2006). Principal components analysis corrects for stratification in genome-wide association studies. Nature Genetics, 38(8), 904–909. 10.1038/ng1847 16862161

[R46] Radloff LS (1977). The CES-D scale: A self-report depression scale for research in the general population. Applied Psychological Measurement, 1(3), 385–401. 10.1177/014662167700100306

[R47] Royston P (2004). Multiple imputation of missing values. STATA Journal, 4(3), 227–241.

[R48] Schmitz LL, Goodwin J, Miao J, Lu Q, Conley D, (2021). The impact of late-career job loss and genetic risk on body mass index: Evidence from variance polygenic scores. Scientific Reports, 11(1), 1–15. 10.1038/s41598-021-86716-y 33828129 PMC8027610

[R49] Schochet PZ (2009). Statistical power for regression discontinuity designs in education evaluations. Journal of Educational and Behavioral Statistics, 34, 238–266. 10.3102/1076998609332748

[R50] Silver RC, Holman EA, Andersen JP, Poulin M, McIntosh DN, & Gil-Rivas V (2013). Mental- and physical-health effects of acute exposure to media images of the September 11, 2001, attacks and the Iraq war. Psychological Science, 24(9), 1623–1634. 10.1177/0956797612460406 23907546

[R51] Simeon D, Yehuda R, Knutelska M, & Schmeidler J (2008). Dissociation versus posttraumatic stress: Cortisol and physiological correlates in adults highly exposed to the World Trade Center attack on 9/11. Psychiatry Research, 161, 325–329. 10.1016/j.psychres.2008.04.021 18930323

[R52] Simon RW (2002). Revisiting the relationships among gender, marital status, and mental health. American Journal of Sociology, 107(4), 1065–1096. 10.1086/339225 12227382

[R53] Strohschein L (2005). Parental divorce and child mental health trajectories. Journal of Marriage and Family, 67(5), 1286–1300. 10.1111/j.1741-3737.2005.00217.x

[R54] Sugie NF, & Turney K (2017). Beyond incarceration: Criminal justice contact and mental health. American Sociological Review, 82(4), 719–743. 10.1177/0003122417713188

[R55] Thistlewaite DL, & Campbell DT (1960). Regression-discontinuity analysis: An alternative to the ex-post facto experiment. Journal of Educational Psychology, 51(6), 309–317.

[R56] Turley P, Walters RK, Maghzian O, Okbay A, Lee JJ, (2018). Multi-trait analysis of genome-wide association summary statistics using MTAG. Nature Genetics, 50, 229–237. 10.1038/s41588-017-0009-4 29292387 PMC5805593

[R57] Young JK, & Beaujean AA (2011). Measuring personality in Wave I of the national longitudinal study of adolescent health. Frontiers in Psychology, 2, 158. 10.3389/fpsyg.2011.00158 21808628 PMC3139206

[R58] Young AI, Benonisdottir S, Przeworski M, & Kong A (2019). Deconstructing the sources of genotype-phenotype associations in humans. Science, 365(6460), 1396–1400. 10.1126/science.aax3710 31604265 PMC6894903

