# Supplementary material for: Detecting genetic heterogeneities in response to trauma: The case of 9/11
Source: SSM Ment Health. Author manuscript; Available in PMC 2023 Jan 20. (PMC9854267; doi:10.1016/j.ssmmh.2021.100044)
Supplement: 1 [file NIHMS1861028-supplement-1.docx]

Appendix A: Differences in demographic characteristics and phenotypes by ancestry groups

Table A1: Differences between ancestry groups

|  | Europe | Africa | Asia | Hispanic |
| --- | --- | --- | --- | --- |
| CES-D score in Wave III | 4.244 | 5.202 | 5.171 | 5.030 |
| Race |  |  |  |  |
| White | 0.003 | 0.984 | 0.969 | 0.277 |
| Non-white | 0.997 | 0.016 | 0.031 | 0.724 |
| Age | 21.82 | 21.86 | 22.35 | 22.17 |
| Sex |  |  |  |  |
| Male | 0.456 | 0.421 | 0.539 | 0.520 |
| Female | 0.544 | 0.579 | 0.461 | 0.480 |
| Mother’s educational attainment |  |  |  |  |
| Below high school | 0.106 | 0.182 | 0.130 | 0.357 |
| High school | 0.326 | 0.299 | 0.241 | 0.275 |
| Some college | 0.314 | 0.301 | 0.244 | 0.235 |
| College graduate | 0.254 | 0.218 | 0.385 | 0.133 |
| PVT score in Wave I | 105.07 | 94.21 | 99.00 | 96.00 |
| Family income | 51.36 | 34.06 | 51.10 | 39.58 |
| CES-D score in Wave I | 10.52 | 12.49 | 13.04 | 12.32 |
| Neuroticism | 11.44 | 10.54 | 12.19 | 11.70 |
| Extraversion | 7.08 | 7.23 | 7.11 | 7.26 |
| Conscientious | 9.01 | 8.35 | 8.43 | 8.67 |
|  |  |  |  |  |
| Observations | 4,726 | 1,628 | 386 | 810 |
| Proportion | 0.626 | 0.216 | 0.051 | 0.107 |

Note: Estimated with multiply imputed datasets (n = 20). Standard deviation of continuous measures are presented in parentheses.

Appendix B: Methodological details for variance depression polygenic score

We estimated SNP effects on the phenotypic variability of depression using data from UK Biobank (UKB), and constructed a variance polygenic score for depression in Add Health. Following previous studies [1,2], we defined depression for each UKB participant by taking the average of non-missing items from four measures of depression, including frequency of depressed mood in the last 2 weeks (data field 2050), frequency of unenthusiasm or disinterest in the last 2 weeks (data field 2060), frequency of tenseness or restlessness in the last 2 weeks, (data field 2070), and frequency of tiredness or lethargy in the last two weeks (data field 2080). We removed UKB participants who satisfy any of the following conditions: recommended by UKB to be removed from the analysis, withdrew from UKB, have conflicting genetically-inferred and self-reported sex, not of European ancestry, have missing data in all four data fields we used to define the depression phenotype. We also removed genetically related samples given by KING [3]. Our total analytical sample size is 374,770.

We then performed a genome-wide variance quantitative trait locus (vQTL) analysis using the QUAIL approach [4] with control variables of sex, year of birth, genotyping array, and the first 20 genetic principal components. Variants that had missing call rates of above 0.01, had a minor allele frequency of below 0.01, or had a Hardy Weinberg equilibrium test p-value of below 1.0e-06 were removed. After these quality controls, 6,995,781 SNPs remained in the vQTL summary statistics. We clumped SNPs using 1,000 Genomes Project Phase III European samples as the reference to remove SNPs with strong linkage disequilibrium (LD). The LD window size of 1 Megabase (Mb) and a pairwise r2 threshold of 0.1 were used. We did not use p-value thresholding to select variants. We applied variance effect sizes estimated from UKB to obtain variance depression polygenic score in Add Health. The depression variance polygenic scores were calculated using PRSice-2 [5].

**References**

[1] Elovainio, M., Hakulinen, C., Pulkki-Råback, L., Virtanen, M., Josefsson, K., Jokela, M., Vahtera, J., Kivimäki, M., Contribution of risk factors to excess mortality in isolated and lonely individuals: an analysis of data from the UK Biobank cohort study. Lancet Public Heal. 2, 2017, e260–e266. https://doi.org/10.1016/S2468-2667(17)30075-0

[2] Niedzwiedz, C.L., Robb, K.A., Katikireddi, S.V., Pell, J.P., Smith, D.J., Depressive symptoms, neuroticism, and participation in breast and cervical cancer screening: Cross-sectional and prospective evidence from UK Biobank. Psychooncology. 29, 2020, 381–388. https://doi.org/10.1002/PON.5272

[3] Manichaikul, A., Mychaleckyj, J.C., Rich, S.S., Daly, K., Sale, M., Chen, W.M., Robust relationship inference in genome-wide association studies. Bioinformatics 26, 2010, 2867–2873. https://doi.org/10.1093/BIOINFORMATICS/BTQ559

[4] Miao, J., Lin, Y., Wu, Y., Zheng, B., Schmitz, L.L., Fletcher, J.M., Lu, Q., A quantile integral linear model to quantify genetic effects on phenotypic variability. bioRxiv 2021.04.14.439847. 2021, https://doi.org/10.1101/2021.04.14.439847

[5] Choi, S.W., O’Reilly, P.F., PRSice-2: Polygenic risk score software for biobank-scale data. GigaScience 8(7), 2019, 1–6. https://doi.org/10.1093/gigascience/giz082

Appendix C: Methodological details of a quadratic polynomial regression discontinuity

To assess the sensitivity of the results, we estimate a quadratic polynomial regression discontinuity. The quadratic regression equation for the general discontinuities at the September 11 attacks is as follows:

$$Y_{i}=\alpha+\beta_{1}T_{i}+\beta_{2}r_{i}+\beta_{3}r_{i}^{2}+\beta_{4}(T_{i}*r_{i})+\beta_{5}(T_{i}*r_{i}^{2})+\boldsymbol{\beta}_{\boldsymbol{6}}\boldsymbol{\chi}_{\boldsymbol{i}}+\varepsilon_{i}$$

Similar with the linear equation, *β_1_*, which is our interest, represents the local average causal effect of the September 11 attacks on the CES-D scores. We then expand this equation to examine the genetic heterogeneities. The regression equation is as follows:

$$Y_{i}=\alpha+\beta_{1}T_{i}+\beta_{2}r_{i}+\beta_{3}r_{i}^{2}+\beta_{4}G_{i}+\beta_{5}\left( T_{i}*r_{i} \right)+\beta_{6}\left( T_{i}*r_{i}^{2} \right)+\beta_{7}\left( T_{i}*G_{i} \right)+\beta_{8}\left( r_{i}*G_{i} \right)+\beta_{9}\left( r_{i}^{2}*G_{i} \right)+\beta_{10}\left( T_{i}*r_{i}*G_{i} \right)+\beta_{11}\left( T_{i}*r_{i}^{2}*G_{i} \right)+{\boldsymbol{\beta}_{\boldsymbol{12}}\boldsymbol{PC}}_{\boldsymbol{i}}+{\boldsymbol{\beta}_{\boldsymbol{13}}\boldsymbol{\chi}}_{\boldsymbol{i}}+\varepsilon_{i}$$

where *β_7_*, which is our interest, denotes the difference of the local average causal effect of the September 11 attacks on the CES-D scores.

Appendix D: Results of sensitivity analyses

Table A2: Results of the quadratic regression discontinuity of the depressive symptom after the September 11 attacks

| VARIABLES | Model 1 | Model 2 | Model 3 | Model 4 | Model 5 | Model 6 |  |
| --- | --- | --- | --- | --- | --- | --- | --- |
|  |  |  |  |  |  |  |  |
| Treatment (ref: Before 9/11) | 0.83 | 1.25† | 0.94† | 0.93† | 0.93* | 0.75* |  |
|  | (0.91) | (0.66) | (0.54) | (0.48) | (0.45) | (0.29) |  |
| Interview date | -0.32 | -0.20 | -0.03 | 0.01 | 0.01 | 0.01 |  |
|  | (0.40) | (0.14) | (0.07) | (0.04) | (0.04) | (0.01) |  |
| Interview date squared | -0.02 | -0.01 | -0.00 | 0.00 | 0.00 | 0.00 |  |
|  | (0.05) | (0.01) | (0.00) | (0.00) | (0.00) | (0.00) |  |
| Treatment x Interview date | 1.09* | 0.28 | 0.04 | -0.03 | -0.04 | -0.02 |  |
|  | (0.47) | (0.18) | (0.10) | (0.06) | (0.05) | (0.01) |  |
| Treatment x Interview date squared | -0.07 | 0.00 | -0.00 | 0.00 | 0.00 | -0.00 |  |
|  | (0.05) | (0.01) | (0.00) | (0.00) | (0.00) | (0.00) |  |
| Constant | 2.45 | 2.93 | 4.03* | 4.46** | 4.75** | 5.40*** |  |
|  | (3.34) | (2.38) | (1.90) | (1.67) | (1.54) | (1.02) |  |
|  |  |  |  |  |  |  |  |
| Observations | 563 | 1,134 | 1,639 | 2,027 | 2,318 | 4,726 |  |

Note: Robust standard errors in parentheses. Additional controls not shown. Estimated with multiply imputed datasets (n = 20).

Model 1, 2, 3, 4, and 5 has bandwidths of 10 days, 20 days, 30 days, 40 days, and 50 days, respectively.
Model 6 includes all observations in our analytical sample. *** p<0.001, ** p<0.01, *p<0.05, † p<0.1

(cont. Table A2, mean depression polygenic score)

| VARIABLES | Model 1 | Model 2 | Model 3 | Model 4 | Model 5 | Model 6 |
| --- | --- | --- | --- | --- | --- | --- |
| Treatment (ref: Before 9/11) | 1.03 | 1.97* | 1.45* | 1.28* | 1.29* | 0.90* |
|  | (1.10) | (0.83) | (0.71) | (0.65) | (0.60) | (0.40) |
| Interview date | -0.33 | -0.42* | -0.10 | -0.00 | 0.00 | 0.00 |
|  | (0.51) | (0.18) | (0.08) | (0.05) | (0.05) | (0.02) |
| Treatment x Interview date | 1.37* | 0.57* | 0.12 | -0.03 | -0.04 | -0.02 |
|  | (0.63) | (0.24) | (0.13) | (0.09) | (0.07) | (0.02) |
| Interview date squared | -0.00 | -0.02* | -0.00 | -0.00 | -0.00 | 0.00 |
|  | (0.06) | (0.01) | (0.00) | (0.00) | (0.00) | (0.00) |
| Treatment x Interview date squared | -0.11 | 0.01 | 0.00 | 0.00 | 0.00 | 0.00 |
|  | (0.07) | (0.01) | (0.00) | (0.00) | (0.00) | (0.00) |
| Mean depression PGS (ref: Low) | 1.82 | 2.06* | 1.18 | 0.69 | 0.69 | 0.58 |
|  | (1.60) | (1.03) | (0.78) | (0.68) | (0.65) | (0.48) |
| Treatment x Mean depression PGS | -0.79 | -1.61 | -1.01 | -0.64 | -0.68 | -0.27 |
|  | (1.76) | (1.27) | (1.06) | (0.95) | (0.88) | (0.58) |
| Interview date x Mean depression PGS | 0.21 | 0.50† | 0.17 | 0.02 | 0.02 | 0.00 |
|  | (0.78) | (0.27) | (0.13) | (0.09) | (0.07) | (0.03) |
| Interview date squared x Mean depression PGS | -0.02 | 0.03† | 0.01 | 0.00 | 0.00 | 0.00 |
|  | (0.09) | (0.01) | (0.00) | (0.00) | (0.00) | (0.00) |
| Treatment x Interview date x Mean depression PGS | -0.74 | -0.62† | -0.19 | -0.03 | -0.01 | -0.00 |
|  | (0.95) | (0.36) | (0.20) | (0.13) | (0.10) | (0.03) |
| Treatment x Interview date squared x Mean depression PGS | 0.07 | -0.02 | -0.01 | -0.00 | -0.00 | -0.00 |
|  | (0.11) | (0.02) | (0.01) | (0.00) | (0.00) | (0.00) |
| Standardized variance depression PGS | 0.16 | 0.22† | 0.18† | 0.14 | 0.12 | 0.17** |
|  | (0.16) | (0.13) | (0.11) | (0.09) | (0.09) | (0.06) |
| Constant | 1.50 | 1.84 | 3.47† | 4.09* | 4.40** | 5.07*** |
|  | (3.17) | (2.28) | (1.85) | (1.65) | (1.52) | (1.03) |
|  |  |  |  |  |  |  |
| Observations | 563 | 1,134 | 1,639 | 2,027 | 2,318 | 4,726 |

Note: Robust standard errors in parentheses. Additional controls not shown. Estimated with multiply imputed datasets (n = 20).

Model 1, 2, 3, 4, and 5 has bandwidths of 10 days, 20 days, 30 days, 40 days, and 50 days, respectively.
Model 6 includes all observations in our analytical sample. *** p<0.001, ** p<0.01, *p<0.05, † p<0.1

(cont. Table A2, variance depression polygenic score)

| VARIABLES | Model 1 | Model 2 | Model 3 | Model 4 | Model 5 | Model 6 |
| --- | --- | --- | --- | --- | --- | --- |
| Treatment (ref: Before 9/11) | 1.49 | 1.13 | 0.31 | 0.10 | 0.04 | 0.32 |
|  | (1.14) | (0.84) | (0.70) | (0.64) | (0.60) | (0.41) |
| Interview date | -0.72 | -0.36* | -0.03 | 0.02 | 0.03 | 0.00 |
|  | (0.53) | (0.18) | (0.09) | (0.06) | (0.05) | (0.02) |
| Treatment x Interview date | 1.23† | 0.40† | 0.04 | -0.03 | -0.03 | -0.01 |
|  | (0.64) | (0.23) | (0.12) | (0.08) | (0.07) | (0.02) |
| Interview date squared | -0.05 | -0.02* | -0.00 | 0.00 | 0.00 | 0.00 |
|  | (0.06) | (0.01) | (0.00) | (0.00) | (0.00) | (0.00) |
| Treatment x Interview date squared | -0.01 | 0.02 | 0.00 | -0.00 | -0.00 | 0.00 |
|  | (0.07) | (0.01) | (0.00) | (0.00) | (0.00) | (0.00) |
| Variance depression PGS (ref: Low) | 1.54 | 0.74 | -0.04 | -0.23 | -0.28 | -0.01 |
|  | (1.43) | (0.98) | (0.77) | (0.69) | (0.66) | (0.48) |
| Treatment x Variance depression PGS | -1.21 | 0.38 | 1.41 | 1.82† | 1.93* | 0.88 |
|  | (1.72) | (1.30) | (1.09) | (0.97) | (0.90) | (0.58) |
| Interview date x Variance depression PGS | 0.75 | 0.33 | 0.02 | -0.03 | -0.04 | 0.00 |
|  | (0.75) | (0.27) | (0.13) | (0.09) | (0.08) | (0.03) |
| Interview date squared x Variance depression PGS | 0.06 | 0.02 | 0.00 | -0.00 | -0.00 | 0.00 |
|  | (0.09) | (0.01) | (0.00) | (0.00) | (0.00) | (0.00) |
| Treatment x Interview date x Variance depression PGS | -0.24 | -0.25 | -0.02 | -0.04 | -0.05 | -0.01 |
|  | (0.89) | (0.35) | (0.19) | (0.13) | (0.10) | (0.03) |
| Treatment x Interview date squared x Variance depression PGS | -0.11 | -0.03 | -0.00 | 0.00 | 0.00 | -0.00 |
|  | (0.10) | (0.02) | (0.01) | (0.00) | (0.00) | (0.00) |
| Standardized mean depression PGS | 0.10 | 0.09 | 0.15 | 0.17† | 0.20* | 0.21*** |
|  | (0.16) | (0.12) | (0.10) | (0.09) | (0.08) | (0.06) |
| Constant | 1.83 | 2.49 | 3.92* | 4.44** | 4.74** | 5.22*** |
|  | (3.36) | (2.37) | (1.91) | (1.69) | (1.56) | (1.05) |
|  |  |  |  |  |  |  |
| Observations | 563 | 1,134 | 1,639 | 2,027 | 2,318 | 4,726 |

Note: Robust standard errors in parentheses. Additional controls not shown. Estimated with multiply imputed datasets (n = 20).

Model 1, 2, 3, 4, and 5 has bandwidths of 10 days, 20 days, 30 days, 40 days, and 50 days, respectively.
Model 6 includes all observations in our analytical sample. *** p<0.001, ** p<0.01, *p<0.05, † p<0.1

Table A3: Results of regression discontinuity of the log-transformed depressive symptom after the September 11 attacks

| VARIABLES | Model 1 | Model 2 | Model 3 | Model 4 | Model 5 | Model 6 |
| --- | --- | --- | --- | --- | --- | --- |
|  |  |  |  |  |  |  |
| Treatment (ref: Before 9/11) |  |  |  |  |  |  |
| Post 9/11 | 0.34** | 0.25** | 0.24** | 0.22*** | 0.21*** | 0.13** |
|  | (0.13) | (0.09) | (0.07) | (0.07) | (0.06) | (0.04) |
| Interview date | -0.03 | -0.00 | 0.00 | 0.00 | 0.00 | 0.00 |
|  | (0.02) | (0.01) | (0.00) | (0.00) | (0.00) | (0.00) |
| Treatment x Interview date | 0.03 | -0.00 | -0.01† | -0.01* | -0.01* | -0.00 |
|  | (0.03) | (0.01) | (0.01) | (0.00) | (0.00) | (0.00) |
| Constant | 0.89 | 1.17** | 1.33*** | 1.40*** | 1.48*** | 1.58*** |
|  | (0.62) | (0.43) | (0.35) | (0.31) | (0.29) | (0.20) |
|  |  |  |  |  |  |  |
| Observations | 563 | 1,134 | 1,639 | 2,027 | 2,318 | 4,726 |

Note: Robust standard errors in parentheses. Additional controls not shown. Estimated with multiply imputed datasets (n = 20).

Model 1, 2, 3, 4, and 5 has bandwidths of 10 days, 20 days, 30 days, 40 days, and 50 days, respectively.
Model 6 includes all observations in our analytical sample. *** p<0.001, ** p<0.01, *p<0.05, † p<0.1

(Table A3 cont., mean depression polygenic score)

| VARIABLES | Model 1 | Model 2 | Model 3 | Model 4 | Model 5 | Model 6 |
| --- | --- | --- | --- | --- | --- | --- |
|  |  |  |  |  |  |  |
| Treatment (ref: Before 9/11) | 0.36* | 0.30* | 0.26* | 0.26** | 0.25** | 0.15* |
|  | (0.17) | (0.13) | (0.11) | (0.10) | (0.09) | (0.06) |
| Interview date | -0.04 | -0.01 | 0.00 | 0.00 | 0.00 | 0.00 |
|  | (0.03) | (0.01) | (0.00) | (0.00) | (0.00) | (0.00) |
| Treatment x Interview date | 0.05 | -0.00 | -0.01† | -0.01* | -0.01* | -0.00 |
|  | (0.04) | (0.01) | (0.01) | (0.01) | (0.00) | (0.00) |
| Mean depression PGS (ref: Low) | 0.19 | 0.13 | 0.07 | 0.08 | 0.09 | 0.11 |
|  | (0.21) | (0.13) | (0.11) | (0.10) | (0.09) | (0.07) |
| Treatment x Mean depression PGS | -0.05 | -0.08 | -0.03 | -0.06 | -0.06 | -0.03 |
|  | (0.25) | (0.17) | (0.15) | (0.13) | (0.12) | (0.08) |
| Interview date x Mean depression PGS | 0.02 | 0.00 | -0.01 | -0.00 | -0.00 | -0.00 |
|  | (0.04) | (0.01) | (0.01) | (0.01) | (0.00) | (0.00) |
| Treatment x Interview date x Mean depression PGS | -0.04 | -0.01 | 0.01 | 0.01 | 0.00 | 0.00 |
|  | (0.05) | (0.02) | (0.01) | (0.01) | (0.01) | (0.00) |
| Standardized variance depression PGS | 0.04 | 0.05† | 0.04† | 0.03 | 0.03 | 0.04*** |
|  | (0.03) | (0.02) | (0.02) | (0.02) | (0.02) | (0.01) |
| Constant | 0.77 | 1.10* | 1.29*** | 1.36*** | 1.43*** | 1.51*** |
|  | (0.62) | (0.42) | (0.35) | (0.31) | (0.29) | (0.20) |
|  |  |  |  |  |  |  |
| Observations | 563 | 1,134 | 1,639 | 2,027 | 2,318 | 4,726 |

Note: Robust standard errors in parentheses. Additional controls not shown. Estimated with multiply imputed datasets (n = 20).

Model 1, 2, 3, 4, and 5 has bandwidths of 10 days, 20 days, 30 days, 40 days, and 50 days, respectively.
Model 6 includes all observations in our analytical sample. *** p<0.001, ** p<0.01, *p<0.05, † p<0.1

(Table A3 cont., variance depression polygenic score)

| VARIABLES | Model 1 | Model 2 | Model 3 | Model 4 | Model 5 | Model 6 |
| --- | --- | --- | --- | --- | --- | --- |
|  |  |  |  |  |  |  |
| Treatment (ref: Before 9/11) | 0.38* | 0.17 | 0.12 | 0.12 | 0.12 | 0.05 |
|  | (0.18) | (0.12) | (0.10) | (0.09) | (0.09) | (0.06) |
| Interview date | -0.05 | -0.00 | 0.00 | 0.00 | 0.00 | 0.00 |
|  | (0.03) | (0.01) | (0.01) | (0.00) | (0.00) | (0.00) |
| Treatment x Interview date | 0.04 | -0.01 | -0.01 | -0.01 | -0.00 | -0.00 |
|  | (0.04) | (0.01) | (0.01) | (0.00) | (0.00) | (0.00) |
| Variance depression PGS (ref: Low) | 0.13 | -0.02 | -0.01 | -0.00 | 0.01 | -0.00 |
|  | (0.20) | (0.13) | (0.11) | (0.10) | (0.09) | (0.07) |
| Treatment x Variance depression PGS | -0.06 | 0.19 | 0.25† | 0.23† | 0.20 | 0.15† |
|  | (0.26) | (0.18) | (0.15) | (0.13) | (0.12) | (0.08) |
| Interview date x Variance depression PGS | 0.04 | -0.00 | -0.00 | -0.00 | -0.00 | -0.00 |
|  | (0.04) | (0.01) | (0.01) | (0.01) | (0.00) | (0.00) |
| Treatment x Interview date x Variance depression PGS | -0.01 | 0.01 | -0.00 | -0.01 | -0.00 | 0.00 |
|  | (0.05) | (0.02) | (0.01) | (0.01) | (0.01) | (0.00) |
| Standardized mean depression PGS | 0.02 | 0.02 | 0.03† | 0.04* | 0.04** | 0.04*** |
|  | (0.03) | (0.02) | (0.02) | (0.02) | (0.02) | (0.01) |
| Constant | 0.83 | 1.16** | 1.31*** | 1.37*** | 1.44*** | 1.54*** |
|  | (0.62) | (0.43) | (0.35) | (0.31) | (0.29) | (0.20) |
|  |  |  |  |  |  |  |
| Observations | 563 | 1,134 | 1,639 | 2,027 | 2,318 | 4,726 |

Note: Robust standard errors in parentheses. Additional controls not shown. Estimated with multiply imputed datasets (n = 20).

Model 1, 2, 3, 4, and 5 has bandwidths of 10 days, 20 days, 30 days, 40 days, and 50 days, respectively.
Model 6 includes all observations in our analytical sample. *** p<0.001, ** p<0.01, *p<0.05, † p<0.1

Table A4: Results of regression discontinuity of the square-rooted depressive symptom after the September 11 attacks

| VARIABLES | Model 1 | Model 2 | Model 3 | Model 4 | Model 5 | Model 6 |
| --- | --- | --- | --- | --- | --- | --- |
|  |  |  |  |  |  |  |
| Treatment (ref: Before 9/11) | 0.44* | 0.32** | 0.30** | 0.28** | 0.27*** | 0.17** |
|  | (0.17) | (0.12) | (0.10) | (0.09) | (0.08) | (0.06) |
| Interview date | -0.04 | -0.00 | 0.00 | 0.00 | 0.00 | 0.00 |
|  | (0.03) | (0.01) | (0.00) | (0.00) | (0.00) | (0.00) |
| Treatment x Interview date | 0.04 | -0.00 | -0.01† | -0.01* | -0.01* | -0.00 |
|  | (0.04) | (0.01) | (0.01) | (0.00) | (0.00) | (0.00) |
| Constant | 1.15 | 1.50** | 1.71*** | 1.79*** | 1.89*** | 2.03*** |
|  | (0.82) | (0.56) | (0.46) | (0.41) | (0.38) | (0.26) |
|  |  |  |  |  |  |  |
| Observations | 563 | 1,134 | 1,639 | 2,027 | 2,318 | 4,726 |

Note: Robust standard errors in parentheses. Additional controls not shown. Estimated with multiply imputed datasets (n = 20).

Model 1, 2, 3, 4, and 5 has bandwidths of 10 days, 20 days, 30 days, 40 days, and 50 days, respectively.
Model 6 includes all observations in our analytical sample. *** p<0.001, ** p<0.01, *p<0.05, † p<0.1

(Table A4 cont., mean depression polygenic score)

| VARIABLES | Model 1 | Model 2 | Model 3 | Model 4 | Model 5 | Model 6 |
| --- | --- | --- | --- | --- | --- | --- |
|  |  |  |  |  |  |  |
| Treatment (ref: Before 9/11) | 0.49* | 0.39* | 0.34* | 0.34** | 0.32** | 0.19* |
|  | (0.22) | (0.16) | (0.14) | (0.13) | (0.12) | (0.08) |
| Interview date | -0.06 | -0.01 | 0.00 | 0.00 | 0.00 | 0.00 |
|  | (0.04) | (0.01) | (0.01) | (0.00) | (0.00) | (0.00) |
| Post 9/11 x Interview date | 0.07 | 0.00 | -0.02† | -0.01* | -0.01* | -0.00 |
|  | (0.05) | (0.02) | (0.01) | (0.01) | (0.01) | (0.00) |
| Mean depression PGS (ref: Low) | 0.31 | 0.18 | 0.10 | 0.11 | 0.13 | 0.15 |
|  | (0.27) | (0.17) | (0.14) | (0.13) | (0.12) | (0.09) |
| Treatment x Mean depression PGS | -0.15 | -0.13 | -0.06 | -0.09 | -0.10 | -0.04 |
|  | (0.32) | (0.23) | (0.20) | (0.17) | (0.16) | (0.11) |
| Interview date x Mean depression PGS | 0.04 | 0.01 | -0.01 | -0.00 | -0.00 | -0.00 |
|  | (0.06) | (0.02) | (0.01) | (0.01) | (0.01) | (0.00) |
| Treatment x Interview date x Mean depression PGS | -0.07 | -0.01 | 0.01 | 0.01 | 0.01 | -0.00 |
|  | (0.07) | (0.02) | (0.01) | (0.01) | (0.01) | (0.00) |
| Standardized variance depression PGS | 0.05 | 0.06† | 0.05† | 0.04 | 0.03 | 0.05*** |
|  | (0.04) | (0.03) | (0.03) | (0.02) | (0.02) | (0.02) |
| Constant | 0.96 | 1.39* | 1.65*** | 1.73*** | 1.82*** | 1.94*** |
|  | (0.81) | (0.56) | (0.46) | (0.41) | (0.38) | (0.26) |
|  |  |  |  |  |  |  |
| Observations | 563 | 1,134 | 1,639 | 2,027 | 2,318 | 4,726 |

Note: Robust standard errors in parentheses. Additional controls not shown. Estimated with multiply imputed datasets (n = 20).

Model 1, 2, 3, 4, and 5 has bandwidths of 10 days, 20 days, 30 days, 40 days, and 50 days, respectively.
Model 6 includes all observations in our analytical sample. *** p<0.001, ** p<0.01, *p<0.05, † p<0.1

(Table A4 cont., variance depression polygenic score)

| VARIABLES | Model 1 | Model 2 | Model 3 | Model 4 | Model 5 | Model 6 |
| --- | --- | --- | --- | --- | --- | --- |
|  |  |  |  |  |  |  |
| Treatment (ref: Before 9/11) | 0.49* | 0.20 | 0.15 | 0.14 | 0.15 | 0.07 |
|  | (0.23) | (0.16) | (0.14) | (0.12) | (0.11) | (0.08) |
| Interview date | -0.07† | -0.00 | 0.00 | 0.00 | 0.00 | 0.00 |
|  | (0.04) | (0.01) | (0.01) | (0.00) | (0.00) | (0.00) |
| Post 9/11 x Interview date | 0.06 | -0.01 | -0.01 | -0.01 | -0.01 | -0.00 |
|  | (0.05) | (0.02) | (0.01) | (0.01) | (0.01) | (0.00) |
| Variance depression PGS (ref: Low) | 0.21 | -0.01 | -0.01 | 0.00 | 0.01 | -0.00 |
|  | (0.27) | (0.17) | (0.14) | (0.13) | (0.12) | (0.09) |
| Treatment x Variance depression PGS | -0.10 | 0.26 | 0.33† | 0.31† | 0.27† | 0.20† |
|  | (0.34) | (0.23) | (0.19) | (0.17) | (0.16) | (0.11) |
| Interview date x Variance depression PGS | 0.06 | -0.00 | -0.00 | -0.00 | 0.00 | -0.00 |
|  | (0.06) | (0.02) | (0.01) | (0.01) | (0.01) | (0.00) |
| Treatment x Interview date x Variance depression PGS | -0.02 | 0.00 | -0.01 | -0.01 | -0.01 | -0.00 |
|  | (0.07) | (0.02) | (0.01) | (0.01) | (0.01) | (0.00) |
| Standardized mean depression PGS | 0.02 | 0.03 | 0.04† | 0.05* | 0.06** | 0.05*** |
|  | (0.04) | (0.03) | (0.03) | (0.02) | (0.02) | (0.02) |
| Constant | 1.05 | 1.48** | 1.67*** | 1.75*** | 1.84*** | 1.98*** |
|  | (0.82) | (0.57) | (0.47) | (0.41) | (0.38) | (0.26) |
|  |  |  |  |  |  |  |
| Observations | 563 | 1,134 | 1,639 | 2,027 | 2,318 | 4,726 |

Note: Robust standard errors in parentheses. Additional controls not shown. Estimated with multiply imputed datasets (n = 20).

Model 1, 2, 3, 4, and 5 has bandwidths of 10 days, 20 days, 30 days, 40 days, and 50 days, respectively.
Model 6 includes all observations in our analytical sample. *** p<0.001, ** p<0.01, *p<0.05, † p<0.1

Appendix E: Power calculation for the RDD and the interaction between the treatment and PGS

Although the RDD is an established causal inference technique, it has been acknowledged that the RDD requires larger samples than experimental studies [1]. This raises a concern that statistical power in the RDD in the current study may be substantially low and the possibility of false positive. To evaluate the validity of the RDD, we used the RDD power calculator in Stata to calculate statistical power for the treatment effect [2]. The summary for the results of power calculation is available in Table A5.

**References**

[1] Schochet, P. Z. (2009). Statistical power for regression discontinuity designs in education evaluations. *Journal of Educational and Behavioral Statistics*, *34*(2), 238–266. https://doi.org/10.3102/1076998609332748

[2] Cattaneo, M. D., Titiunik, R., & Vazquez-Bare, G. (2019). Power calculations for regression-discontinuity designs: *The Stata Journal*, 19(1), 210–245. https://doi.org/10.1177/1536867X19830919

Table A5: Summary of calculated statistical power

| Bandwidths | Statistical Power |
| --- | --- |
| 10 days | 0.610 |
| 20 days | 0.885 |
| 30 days | 0.852 |
| 40 days | 0.726 |
| 50 days | 0.661 |
| Full model | 0.302 |

Note: Statistical power was calculated by RDD calculator created by Cattaneo et al. (2019).

Appendix F: Results with the standardized depression polygenic scores

Table A6: Results of regression discontinuity of the depressive symptom after the September 11 attacks by the mean depression polygenic score

| VARIABLES | Model 1 | Model 2 | Model 3 | Model 4 | Model 5 | Model 6 |
| --- | --- | --- | --- | --- | --- | --- |
|  |  |  |  |  |  |  |
| Treatment (ref: Before 9/11) | 1.42* | 1.05* | 1.03** | 0.96** | 0.92** | 0.64** |
|  | (0.65) | (0.46) | (0.39) | (0.34) | (0.32) | (0.23) |
| Interview date | -0.15 | -0.00 | 0.00 | 0.00 | 0.00 | 0.00 |
|  | (0.12) | (0.03) | (0.02) | (0.01) | (0.01) | (0.01) |
| Treatment x Interview date | 0.18 | -0.02 | -0.04 | -0.03 | -0.02 | -0.01 |
|  | (0.14) | (0.04) | (0.03) | (0.02) | (0.01) | (0.01) |
| Standardized mean depression PGS | 0.68† | 0.05 | -0.03 | 0.02 | 0.04 | 0.11 |
|  | (0.41) | (0.26) | (0.23) | (0.21) | (0.20) | (0.16) |
| Treatment x Standardized mean depression PGS | -0.56 | 0.01 | 0.11 | 0.04 | 0.03 | 0.11 |
|  | (0.55) | (0.42) | (0.35) | (0.32) | (0.29) | (0.20) |
| Interview date x Standardized mean depression PGS | 0.15 | -0.01 | -0.02 | -0.02 | -0.02 | -0.01 |
|  | (0.09) | (0.03) | (0.02) | (0.01) | (0.01) | (0.01) |
| Treatment x Interview date x Standardized mean depression PGS | -0.16 | 0.01 | 0.03 | 0.02 | 0.02† | 0.01 |
|  | (0.12) | (0.04) | (0.02) | (0.02) | (0.01) | (0.01) |
| Standardized variance depression PGS | 0.19 | 0.23† | 0.18 | 0.13 | 0.11 | 0.15* |
|  | (0.17) | (0.13) | (0.11) | (0.10) | (0.09) | (0.06) |
| Constant | 1.86 | 3.12 | 3.92* | 4.29** | 4.55** | 5.27*** |
|  | (3.29) | (2.28) | (1.86) | (1.64) | (1.50) | (1.01) |
|  |  |  |  |  |  |  |
| Observations | 563 | 1,134 | 1,639 | 2,027 | 2,318 | 4,726 |

Note: Robust standard errors in parentheses. Additional controls not shown. Estimated with multiply imputed datasets (n = 20).

*** p<0.001, ** p<0.01, *p<0.05, † p<0.1

Table A7: Results of regression discontinuity of the depressive symptom after the September 11 attacks by the variance depression polygenic score

| VARIABLES | Model 1 | Model 2 | Model 3 | Model 4 | Model 5 | Model 6 |
| --- | --- | --- | --- | --- | --- | --- |
|  |  |  |  |  |  |  |
| Treatment (ref: Before 9/11) | 1.50* | 1.06* | 1.04** | 0.98** | 0.94** | 0.63** |
|  | (0.65) | (0.46) | (0.39) | (0.35) | (0.32) | (0.23) |
| Interview date | -0.16 | -0.00 | 0.01 | 0.00 | 0.00 | 0.00 |
|  | (0.12) | (0.03) | (0.02) | (0.01) | (0.01) | (0.01) |
| Treatment x Interview date | 0.19 | -0.03 | -0.04 | -0.03 | -0.02† | -0.01 |
|  | (0.14) | (0.05) | (0.03) | (0.02) | (0.01) | (0.01) |
| Standardized variance depression PGS (ref: Low) | 0.29 | -0.04 | 0.04 | 0.08 | 0.10 | 0.03 |
|  | (0.43) | (0.29) | (0.25) | (0.23) | (0.21) | (0.17) |
| Treatment x Standardized variance depression PGS | -0.10 | 0.56 | 0.52 | 0.37 | 0.28 | 0.22 |
|  | (0.58) | (0.44) | (0.37) | (0.33) | (0.30) | (0.21) |
| Interview date x Standardized variance depression PGS | 0.07 | -0.02 | -0.00 | 0.00 | 0.01 | 0.00 |
|  | (0.10) | (0.03) | (0.02) | (0.01) | (0.01) | (0.01) |
| Treatment x Interview date x Standardized variance depression PGS | -0.01 | -0.00 | -0.02 | -0.02 | -0.02 | -0.00 |
|  | (0.12) | (0.04) | (0.02) | (0.02) | (0.01) | (0.01) |
| Standardized mean depression PGS | 0.09 | 0.10 | 0.16 | 0.18* | 0.21* | 0.20*** |
|  | (0.16) | (0.12) | (0.10) | (0.09) | (0.08) | (0.06) |
| Constant | 1.84 | 3.24 | 4.01* | 4.36** | 4.62** | 5.31*** |
|  | (3.32) | (2.28) | (1.86) | (1.64) | (1.50) | (1.01) |
|  |  |  |  |  |  |  |
| Observations | 563 | 1,134 | 1,639 | 2,027 | 2,318 | 4,726 |

Note: Robust standard errors in parentheses. Additional controls not shown. Estimated with multiply imputed datasets (n = 20).

*** p<0.001, ** p<0.01, *p<0.05, † p<0.1

Appendix G: Assessment for the potential impact of the measurement error in polygenic score.

Because of the possibility of the presence of the large amount of measurement error in the depression polygenic score, we assessed the potential impact of the measurement error in the depression polygenic score on the treatment effect and the interaction between the treatment and the depression polygenic score under the assumption of the classical measurement error. Specifically, we generated a true model and evaluated to what extent the coefficients of the treatment and the interaction change as the measurement error in the variance depression polygenic score increases. The coefficients in the true model was derived from the result with the bandwidths of 50 days in Table 4. We set these coefficients of 1 for the sake of simplicity. Overall, below is the true model in this simulation:

$$Y_{i}=T_{i}+0.00*r_{i}+0.58*G_{i}-0.03*\left( T_{i}*r_{i} \right)+\left( T_{i}*G_{i} \right)+0.00*\left( r_{i}*G_{i} \right)+0.02*\left( T_{i}*r_{i}*G_{i} \right)+\varepsilon_{i}$$

Figure A1 presents the effect of the measurement error in the depression polygenic score on the coefficients of the treatment and the interaction. Results show that the measurement error in the variance depression polygenic score increases the coefficient of the treatment, whereas the coefficient of the interaction is biased toward zero as the measurement error increases. Looking at the intersection of the dotted line and the solid blue line, the coefficient of the interaction is around 0.30 when the reliability is 0.23 (i.e., 77% of the variance of the depression polygenic score is measurement error). Given that the measurement error in our depression polygenic score is equivalent to that reported in the prior study [1], this suggests that the estimated interaction is attenuated by 70%.

**References**

[1] Turley, P., Walters, R.K., Maghzian, O., Okbay, A., Lee, J.J. et al., Multi-trait analysis of genome-wide association summary statistics using MTAG. Nat. Genet. 50, 2018, 229–237. https://doi.org/10.1038/s41588-017-0009-4

Figure A1: Changes in regression coefficients corresponding to the measurement error in depression polygenic score.


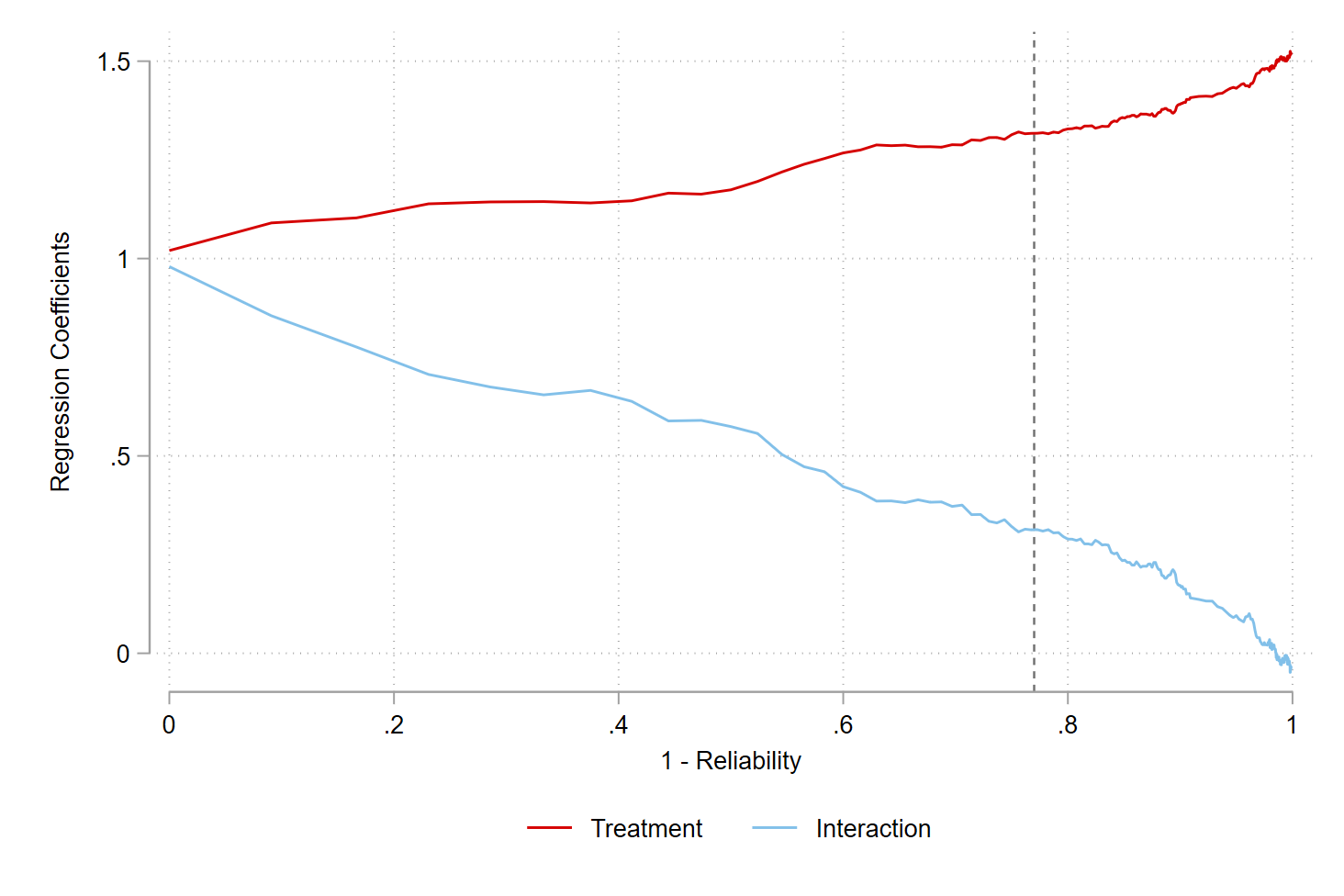


Note: Dotted line represents the reliability of 0.23.
